# Umbilical Cord Pericytes Provide a Viable Alternative to Mesenchymal Stem Cells for Neonatal Vascular Engineering

**DOI:** 10.3389/fcvm.2020.609980

**Published:** 2021-01-21

**Authors:** William Cathery, Ashton Faulkner, Eva Jover, Iker Rodriguez-Arabaolaza, Anita C. Thomas, Elisa Avolio, Massimo Caputo, Paolo Madeddu

**Affiliations:** ^1^Bristol Medical School, Translational Health Sciences, University of Bristol, Bristol, United Kingdom; ^2^School of Biochemistry, University of Bristol, Bristol, United Kingdom; ^3^Cardiovascular Translational Research, Navarrabiomed, Instituto de Investigación Sanitaria de Navarra-IdiSNA, Pamplona, Spain; ^4^Vascular Pathophysiology Area, Centro Nacional de Investigaciones Cardiovasculares Carlos III, Madrid, Spain

**Keywords:** pericytes, tissue engineering, vascular grafts, regenerative medicine, congenital heart disease

## Abstract

Reconstructive surgery of congenital heart disease (CHD) remains inadequate due to the inability of prosthetic grafts to match the somatic growth of pediatric patients. Functionalization of grafts with mesenchymal stem cells (MSCs) may provide a solution. However, MSCs represent a heterogeneous population characterized by wide diversity across different tissue sources. Here we investigated the suitability of umbilical cord pericytes (UCPs) in neonatal vascular engineering. Explant outgrowth followed by immunomagnetic sorting was used to isolate neural/glial antigen 2 (NG2)^+^/CD31^−^ UCPs. Expanded NG2 UCPs showed consistent antigenic phenotype, including expression of mesenchymal and stemness markers, and high proliferation rate. They could be induced to a vascular smooth muscle cell-like phenotype after exposure to differentiation medium, as evidenced by the expression of transgelin and smooth muscle myosin heavy chain. Analysis of cell monolayers and conditioned medium revealed production of extracellular matrix proteins and the secretion of major angiocrine factors, which conferred UCPs with ability to promote endothelial cell migration and tube formation. Decellularized swine-derived grafts were functionalized using UCPs and cultured under static and dynamic flow conditions. UCPs were observed to integrate into the outer layer of the graft and modify the extracellular environment, resulting in improved elasticity and rupture strain in comparison with acellular grafts. These findings demonstrate that a homogeneous pericyte-like population can be efficiently isolated and expanded from human cords and integrated in acellular grafts currently used for repair of CHD. Functional assays suggest that NG2 UCPs may represent a viable option for neonatal tissue engineering applications.

## Introduction

Congenital heart disease (CHD) is the most prevalent congenital abnormality, affecting ~1% of newborns globally, with incidence rate seemingly increasing over the last 50 years (1). Although prognosis has significantly improved, CHD remains one of the primary causes of perinatal mortality, accounting for over 250,000 deaths in 2017 (2). Complex malformations such as Tetralogy of Fallot (ToF) often require immediate surgical correction using a prosthetic graft. Unfortunately, current grafts have limitations and need to be substituted through repeated interventions due to pathologic remodeling and failure (3, 4).

In recent years, engineering of prosthetic grafts with regenerative cells, such as mesenchymal stem cells (MSCs), has emerged as a potential solution (5, 6). However, MSCs represent a heterogeneous population characterized by wide diversity across different tissue sources. Evidence from our group and others have highlighted the potential advantage of using more homogeneous perivascular mesenchymal populations, such as microvascular and adventitial pericytes, for applications of vascular regenerative medicine (7, 8). Moreover, we demonstrated the feasibility of reconstructing the pulmonary artery of piglets using grafts engineered with pericytes immunosorted from cardiac tissue (9). Cardiac pericytes attract endothelial cells (ECs) and produce extracellular matrix (ECM) proteins, which may help to alleviate the limitations of the acellular technology. Nonetheless, the use of heart-derived cells is only compatible with a two-stage intervention—an invasive harvesting followed by implantation—with risks superior to an immediate correction approach (10–12). An alternative cell population from an easily accessible tissue, such as the umbilical cord, would therefore offer obvious advantages over cardiac pericytes.

Perivascular progenitor cells characterized by a myofibroblastic mesenchymal phenotype have been expanded from pre-term umbilical cords using a dissection/culture selection procedure; however, key stemness features were lost when applying the same method to isolate perivascular cells from full-term cords (13). Immunomagnetic sorting provides a more refined technique to isolate target cells from heterogeneous mixtures. To the best of our knowledge, antigenically pure pericytes from umbilical cord of full-term neonates have never been tested for graft engineering.

The present study established a novel method for isolation and expansion of umbilical cord pericytes (UCPs) expressing the neural/glial antigen (NG2). We have characterized the antigenic and secretory phenotype of NG2 UCPs and their ability to differentiate along the vascular smooth muscle cell (VSMC) lineage and modulate angiogenesis *in vitro*. Moreover, we showed that NG2 UCPs can engraft onto a clinically available conduit made of small intestinal submucosa extracellular matrix (CorMatrix®), promoting ECM remodeling and improving the conduit mechanical properties.

## Materials and Methods

### Ethics

Full-term human umbilical cord samples were obtained with informed consent from St. Michael's Hospital, Bristol, UK. Their use throughout the project was approved by the Southwest Research Ethics Committee (license 11/HO107/4) and conformed to the principles outlined in the Declaration of Helsinki.

### Cell Isolation and Culture

Initial isolation of pericytes from human umbilical cord samples was attempted using a protocol previously developed by our group for the isolation of saphenous vein and cardiac pericytes (14, 15). Briefly, umbilical cord sections were enzymatically digested and cell suspensions immunomagnetically sorted using anti-CD31 and anti-CD34 microbeads (Miltenyi Biotech). CD31^−^/CD34^+^ cells were cultured in endothelial growth medium 2 (EGM-2; Promocell) on tissue culture plastic pre-coated with 0.01% (w/v) gelatin (Sigma-Aldrich) and 10 mg/ml fibronectin (Sigma-Aldrich) and maintained at 37°C (5% CO_2_) (14, 15).

**Figure 1:**
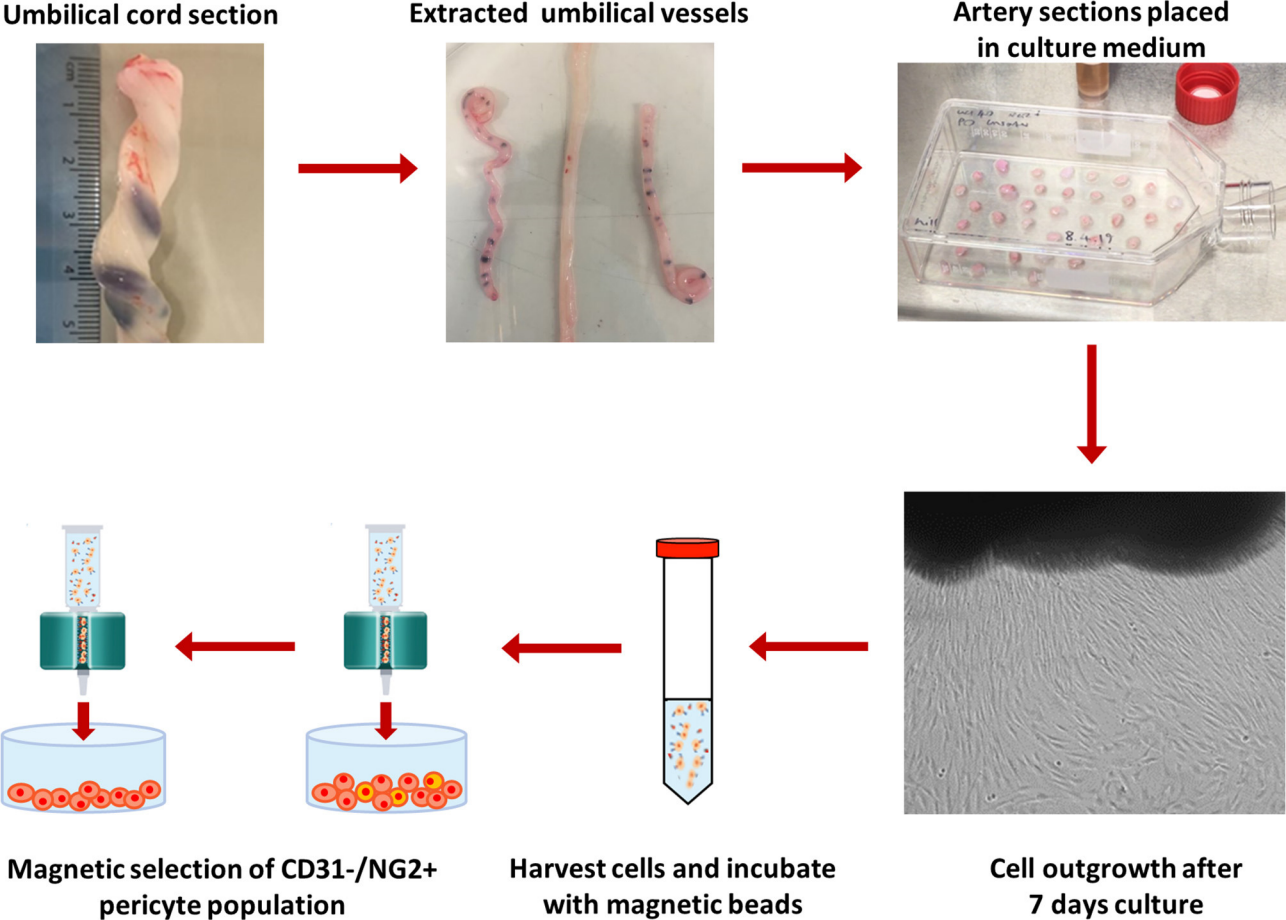
Protocol for isolation of NG2 UCPs. Umbilical vessels were extracted from the cord and the Wharton's Jelly removed. 1–3 mm^2^ sections of the umbilical artery were placed in a flask and cultured in EGM-2. After 7–10 days, the tissue sections were removed, and migrated cells were collected. The cells were sorted to obtain an NG2^+^/CD31^−^ pericyte population using immunomagnetic selection. The purified cell population was cultured in EGM-2 and expanded for further experimentation.

An alternative method for isolation of UCPs was developed, which utilized explant outgrowth instead of enzymatic digestion (Figure 1). The umbilical vessels were extracted from the cord and Wharton's Jelly removed. Sections (1–3 mm^2^) of the umbilical artery were placed in a flask and cultured in EGM-2. After 7–10 days in culture, colonies of cells were observed migrating out from the explants. The explant tissue was removed using sterile forceps, and the migrated cells harvested. This heterogenous cell population was then purified using immunomagnetic selection. Briefly, cells were incubated with anti-CD31 microbeads in column buffer, comprising of 0.5% (w/v) bovine serum albumin (BSA; Sigma-Aldrich) and 2 mM pH8 EDTA (Ambion), according to manufacturer's instructions. The suspension was filtered through a magnetic column, keeping the CD31^−^ cell population, and the selection process was repeated using anti-NG2 beads (Miltenyi Biotech), retaining the NG2^+^ pericyte population. The acquired cell population was then cultured in EGM-2 on pre-coated tissue culture plastic as detailed above and maintained a 37°C (5% CO_2_).

Human Wharton's Jelly MSCs (Promocell) were used for comparative studies with isolated pericytes due to their frequent application in tissue engineering studies (5, 6). MSCs were expanded in mesenchymal stem cell growth medium 2 (Promocell). Human umbilical vein endothelial cells (HUVECs; Lonza) were cultured in EGM-2 on 0.5% (w/v) gelatin coated surfaces. Adult normal human dermal fibroblasts (NHDF; Lonza) were cultured in fibroblast growth medium 2 (Lonza).

All cells were used for experimentation between passages 3 and 7.

### Immunohistochemistry

Tissue samples were collected and fixed in 4% (w/v) buffered paraformaldehyde (PFA; Sigma-Aldrich) for 24 h at 4°C. Samples were then washed in phosphate buffered saline (PBS), placed in 30% (w/v) sucrose for 24 h at 4°C, and embedded in optimal cutting temperature compound (Tissue-Tek® O.C.T., from VWR). Eight-micron sections were cut using a CryoStar NX50 cryostat (Thermo Fisher Scientific) set to −20°C, and mounted onto Superfrost Plus slides (Thermo Fisher Scientific). Slides were hydrated in PBS for 10 min and permeabilized with 0.01% Triton-X100 (Sigma-Aldrich) in PBS for 10 min at 20°C. Non-specific binding sites were blocked using 5% fetal bovine serum (FBS) in PBS for 30 min at 20°C. Slides were triple stained with CD31, CD34, and NG2 primary antibodies (Supplementary Table 1). Cells were incubated with appropriate secondary antibodies (Supplementary Table 2) for 1 h at 20°C, before counterstaining with DAPI and imaging with a Zeiss Observer.Z1 microscope.

### Expansion Capacity and Viability

Cells were seeded in a 6-well plate at a density of 3,000 cells/cm^2^. They were detached on day 4, 5, 6, and 7, and live cells counted after Trypan Blue Solution (Thermo Fisher Scientific) exclusion staining. Doubling time was determined using a growth curve and viability was calculated from the number of live and dead cells.

### Immunocytochemistry

Adherent cells were fixed with 4% PFA for 10 min and blocked with 5% FBS in PBS. For detection of intracellular or transmembrane antigens, cells were permeabilized prior to blocking using 0.1% Triton-X100 (Sigma-Aldrich) in PBS for 10 min at 4°C. Primary antibodies were incubated overnight at 4°C (Supplementary Table 1). Cells were incubated with appropriate secondary antibodies (Supplementary Table 2) for 1 h at 20°C, before counterstaining with DAPI and imaging with a Zeiss Observer.Z1 microscope.

### Flow Cytometry

Cultured cells were harvested and resuspended in staining buffer comprising of 1% (w/v) BSA and 1 mM EDTA pH8 in PBS prior to blocking with Human Fc Receptor Binding Inhibitor. Tubes were prepared with 200,000 cells incubated with 100 μl antibodies in staining buffer at 4°C in the dark for 30 min (Supplementary Table 3). Cells were labeled using Zombie NIR™ viability dye (BioLegend) and dead cells were excluded. Analysis was completed using a NovoCyte Flow Cytometer System (ACE Bioscience) and FlowJo software package. Representative raw histogram data is demonstrated in Supplementary Figure 1.

### Secretome Characterization

Cell conditioned medium (CM) was collected from NG2 UCPs and MSCs, which were cultured for 48 h in serum/serum and vascular endothelial growth factor (VEGF)-free EGM-2 under culture normoxia (20% O_2_) or hypoxia (2% O_2_). This medium was centrifuged to remove detached cells and stored at −80°C until analysis by ELISA, angiogenesis assay or migration assay. The quantities of angiopoietin-1 (ANGPT-1), Angiopoietin-2 (ANGPT-2), stromal derived factor-1 (SDF-1), VEGF-A, and monocyte chemoattractant protein-1 (MCP-1) were determined in CM using specific anti-human ELISA kits (all from R&D Systems) following manufacturer's instructions. Values were normalized by the conditioning time and number of cells at harvest.

### Angiogenesis Assay

The pro-angiogenic potential of cells was evaluated using a co-culture angiogenesis assay as previously described (16, 17). Two different approaches were utilized to investigate if NG2 UCPs pro-angiogenic potential results from their secretome or physical cell-to-cell crosstalk. Briefly, NHDFs were seeded (10,000 cell/cm^2^) in either a 96-well plate [CM (secretome) analysis] or 24-well plate (cell-to-cell contact analysis) and cultured over 5 days until a confluent monolayer had formed. HUVECs were then seeded (10,000 cell/cm^2^) in either CM or control medium (serum/VEGF-free EGM-2) (for CM analysis), or co-seeded with either NG2 UCPs or MSCs (pre-labeled with DsRED using lentiviral transduction; 2,500 cell/cm^2^) in EBM-2 (10% FBS) (for cell-to-cell contact analysis). Following incubation for 6 days, with medium refreshed every 2 days, cells were fixed with 4% PFA and networks were stained with anti-CD31 antibody (1:200; R&D Systems). Images were acquired using a Zeiss Axio Observer.Z1 microscope and analyzed using ImageJ.

### Differentiation to Vascular Smooth Muscle Cells

Differentiation of NG2 UCPs was achieved by incubation with transforming growth factor beta (TGF-β). Cells were cultured in complete EGM-2 until 80% confluent before changing to the differentiation medium, consisting of EGM-2 supplemented with a 2 ng/ml TGF-β1 (Peprotech) dosage, as previously reported (18). EGF and FGF were removed due to their attenuation of TGF-β activity (19). After 15 days incubation with the differentiation medium, replenished every 3 days, cells were harvested for analysis. Undifferentiated cells cultured in complete EGM-2 were used as a negative control for VSMC marker expression.

### Reverse Transcription-Quantitative PCR (RT-qPCR)

Total RNA was extracted from cells using a miRNeasy Mini Kit (Qiagen) and reverse transcribed into first-strand cDNA using a High Capacity RNA-to-cDNA Kit (Life Technologies) according to manufacturer's instructions. Relative mRNA expression was determined by quantitative PCR (qPCR) using TaqMan™ Universal Master Mix II with UNG (Thermo Fisher Scientific) and a QuantStudio 6 Flex Real-Time PCR system (Applied Biosystems). A list of probes used can be found in Supplementary Table 4. Analysis was performed using the 2^−ΔΔCt^ (Livak) method and results normalized to internal housekeeping control gene Ubiquitin C (UBC). A no template control (NTC) was included for analysis.

### Western Blot

Cell lysates were collected in RIPA buffer (Life Technologies) supplemented with 1/100 (v/v) protease inhibitors (Sigma-Aldrich) and 1/50 (v/v) phosphatase inhibitors (Sigma-Aldrich). Protein concentration was determined using Pierce BCA Protein Assay (Thermo Fisher Scientific). Protein samples (10 or 20 μg) were resolved in 8–10% SDS-PAGE gels at 120 V for 90 min and transferred onto 0.22 μm pore size polyvinylidene difluoride (PVDF) membranes (GE Healthcare) using a wet system set to 0.25 A for 60–75 min. Membranes were blocked in 5% non-fat milk in 100 mM Tris, 150 mM NaCl, 0.05% Tween-20 for 1 h at 20°C before incubating with primary antibodies at 4°C overnight (Supplementary Table 1). Appropriate ECL-HRP-conjugated secondary antibodies (Supplementary Table 2) were applied to the membranes for 1 h at 20°C and proteins were detected by enhanced chemiluminescence (ECL) using Amersham™ ECL Reagent (GE Healthcare). A BioRad ChemiDoc MP was used to image the protein bands, which were then semi-quantified by densitometry analysis using ImageJ and normalized to β-Tubulin loading control. Representative western blot images are demonstrated in Supplementary Figure 2.

### Contraction Assay

The contractile properties of differentiated and undifferentiated cells were assessed using a collagen gel contraction assay. Collagen gels (2 mg/ml) were prepared in a 24-well plate (250 μl/cm^2^) using Rat Tail Collagen I (Thermo Fisher Scientific) according to manufacturer's instructions. Cells (50,000/cm^2^) were seeded in the gel, which was left to polymerize for 1 h at 37°C/5% CO_2_ before the addition of the differentiation medium or control medium and incubation for 24 h. After 24 h incubation, cell contraction was assessed using fresh medium supplemented with vasoconstrictor endothelin-1 (0.1 μM, Sigma Aldrich) with or without the myosin ATPase inhibitor 2,3-butanedione monoxime (10 mM, BDM; cell Biolabs). Gels were left for a further 1 h before releasing and conditioning for an additional 24 h. Contraction was quantified using ImageJ by measuring the change in gel area over the course of the experiment.

### Scratch Wound Migration Assay

Confluent HUVEC monolayers were seeded in 0.5% gelatin-coated 96 well-plates (15,000 cells/cm^2^) in EGM-2. A scratch was made using a 10 μl pipette tip. HUVECs were then incubated for 12 h with CM or control medium, which were supplemented with 2 mM hydroxyurea (Sigma-Aldrich) to inhibit proliferation. HUVECs were imaged at 0 and 12 h using a Leica DMi1 inverted microscope and the scratch area quantified using ImageJ.

### ECM Production

To evaluate the cellular production of ECM, the elastin and collagen content of confluent cell monolayers was quantified. Cells were seeded in a 6-well plate (20,000 cells/cm^2^) and conditioned in 2 mL EGM-2. After 3 and 5 days of culture, monolayers and CM were processed for elastin and collagen using a Fastin™ Elastin assay or Sircol™ collagen colorimetric assays (Biocolor) according to manufacturer's instructions. Content was quantified using a GloMax Discover Microplate Reader.

The presence of extracellular proteins was also assessed in decellurized matrix. Briefly, cells were seeded in 96-well plates (20,000 cells/cm^2^) and confluent monolayers decellurized by incubating cells with cold 1 mM EDTA pH8 prepared in PBS for 24 h at 4°C, as described by Pattabhi et al. (20). This process was repeated until all cells were removed, and collagen and fibronectin content was analyzed using immunocytochemistry (Supplementary Table 1).

### ECM Degradation

To assess the ability of cells to degrade the ECM, cell lysates and CM were screened for the presence of active matrix metalloproteinases (MMPs). Briefly, 500,000 cells were seeded in a T25 culture flask (20,000 cells/cm^2^) and grown until confluent. Once cells became confluent, the medium was changed and cells were conditioned for 48 h. CM and cell lysate were harvested and analyzed using a Human MMP Antibody Array-Membrane-ab134004 (abcam) according to manufacturer's instructions. A BioRad ChemiDoc MP was used to image the membranes, which were semi-quantified using ImageJ and normalized to the membrane positive control. Results were normalized to either total protein or total CM volume as appropriate. Representative array membrane images are demonstrated in Supplementary Figure 3. Cell lysates and CM were also analyzed for collagenase activity using a fluorometric Collagenase (Collagen Degradation/Zymography) Assay Kit-ab234624, according to manufacturer's instructions. Total intracellular and extracellular collagenase activity was calculated by scaling measurements according to total protein and CM volume per T25 culture flask.

### Graft Production and Analysis

40 × 30 mm pieces of CorMatrix (CorMatrix Cardiovascular) were cut and positioned in sterile crowns before priming for 24 h in EGM-2 at 37°C/5% CO_2_. NG2 UCPs were then seeded at a density of 20,000/cm^2^ and cultured in fresh medium for a further 5 days under static conditions. Samples were then either analyzed directly or prepared for an additional 7 days dynamic conditioning. For preparation of dynamic conditioning, the pieces of CorMatrix were stitched into the shape of a conduit, with the pericytes facing the external abluminal side of the conduit. The bioreactor system was set up consisting of a Masterflex L/S digital 07528-20 peristaltic pump, tubing and 3DCulture Pro bioreactor chambers. The conduit was then positioned into the bioreactor chamber in fresh EGM-2 and conditioned for a further 7 days (flow rate 24 ml/min). The conduits were then analyzed using the assays described below.

For histological analysis, conduit sections were embedded and probed with αSMA, NG2, or vimentin as detailed in immunohistochemistry method using antibodies listed in Supplementary Tables 1, 2. Hematoxylin and Eosin (H&E), Verhoef's van Geison (EVG), and Mallory's trichrome staining were completed to visualize the structure, elastin and collagen content using a Shandon Varistan 24-4 slide stainer (Thermo Fisher Scientific). Images were acquired using a Zeiss Axio Observer.Z1 microscope.

Cell proliferation was calculated by probing with marker of proliferation Ki-67 (1:100; DAKO) followed by incubation with goat anti-mouse Alexa fluor 488 (Life Technologies) and counterstained with DAPI as per immunocytochemistry method. Proliferation and density of cells was calculated by counting the number of DAPI positive and Ki-67 positive cells. Viability was analyzed using a LIVE/DEAD™ Viability/Cytoxicity Kit (Thermo Fisher Scientific) according to manufacturer's instructions. Cell-seeded scaffolds were counterstained using Hoechst (1:1,000, 10 min; Thermo Fisher Scientific). Viability was calculated by counting the proportion of live to dead cells. Presence of soluble collagen in medium collected after static and dynamic conditioning was assessed using a human Pro-Collagen I alpha 1 ELISA kit (R&D Systems) as per manufacturer's instructions. Results were normalized by conditioning time.

For mechanical analysis, 5 × 20 mm sections were harvested from the graft and stretched lengthwise using an Instron 3343 tensile machine (Illinois Tool Works Inc), set at 10 mm/min. The exact area and length of each section was measured using Vernier calipers, and a stress-strain curve plotted to calculate the Young's modulus and rupture strain. Tests were carried out at 37°C in PBS.

Unseeded controls were included for comparison in all stages of graft production and analysis.

### Statistics

Statistical analysis was performed using GraphPad Prism software. Differences between 2 experimental groups were assessed using student's *t*-test. Differences between more than 2 groups were evaluated using one or two-way analysis of variance (ANOVA), followed by Sidak's *post-hoc* test or Tukey's *post-hoc* test. All data are presented as mean ± standard error of the mean (SEM), and *p* < 0.05 was considered statistically significant.

## Results

### Isolation and Expansion of Pericytes From Umbilical Cord

**Figure 2:**
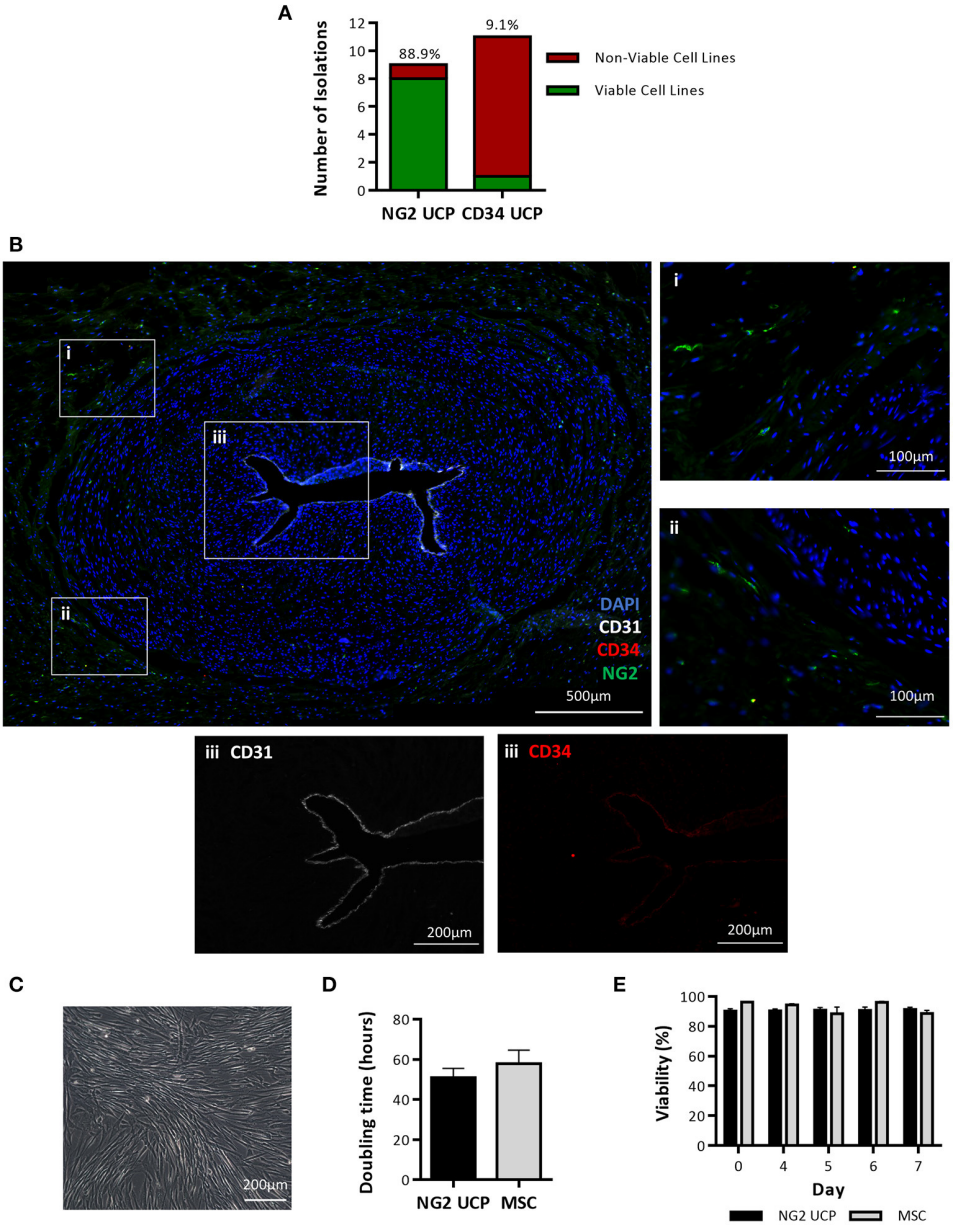
Isolation and expansion of umbilical cord pericytes. **(A)** Successful and unsuccessful cell isolations from the umbilical cord using two immunomagnetic sorting methods based on NG2 and CD34 antigens. The percentage above each column indicates the efficiency of the isolation protocol. **(B)** Immunohistochemical staining of umbilical artery. DAPI, blue; CD31, white; CD34, red; and NG2, green. Inserts in (i, ii) show NG2 positive pericytes negative for CD31 and CD34. Insert III shows CD31 and CD34 expression is restricted to the endothelial layer in the lumen. **(C)** Morphology of NG2 UCPs. **(D)** Doubling time in culture, calculated from cell growth curve. **(E)** Viability analysis of cells in culture. Data represent means (±S.E.M). MSCs and NG2 UCPs, *n* = 3 and 6 biological replicates, respectively.

The first objective of this study was to determine if pericytes could be separated from human umbilical cords using an immunomagnetic beads sorting protocol previously established for extraction of CD31^−^/CD34^+^ pericytes from saphenous vein and cardiac tissue (14, 15). Using this method, a total of 11 umbilical cord isolations were attempted, however only one viable cell line of CD34^+^ pericytes (CD34 UCPs) was successfully expanded, resulting in an isolation efficiency of just 9.1% (Figure 2A). To improve the isolation of pericytes from umbilical cord, an alternative methodology was designed, which incorporated explant outgrowth from cultured umbilical artery, followed by an immunomagnetic selection of CD31^−^/NG2^+^ cells, as detailed in Methods. A total of 9 isolations were attempted, resulting in eight viable NG2^+^ umbilical cord pericyte (NG2 UCP) populations (Figure 2A). Immunohistochemical analysis of the umbilical cord confirmed no CD31^−^/CD34^+^ pericytes were present within the tissue; however, only sporadic staining of NG2 UCPs was detected (Figure 2B). These data indicate that the native antigenic characteristics of *bona fide* perivascular cord pericytes differ from that of other tissue sources, thus requiring substantial modification of the isolation protocol.

Cultured NG2 UCPs displayed a spindle-shaped morphology, typical of pericytes (Figure 2C). The expansion capacity of NG2 UCPs was similar to that of cord MSCs, with a population doubling time of 50.9 ± 4.7 and 57.9 ± 6.7 h, respectively (Figure 2D). As a result of the high initial yield of cells following isolation, we could expand >10 million NG2 UCPs by passage 5 within a 6-week period. The viability of cells also remained high throughout culture and was not significantly different between NG2 UCPs and MSCs (Figure 2E).

### Antigenic Characteristics of NG2 UCPs

**Figure 3:**
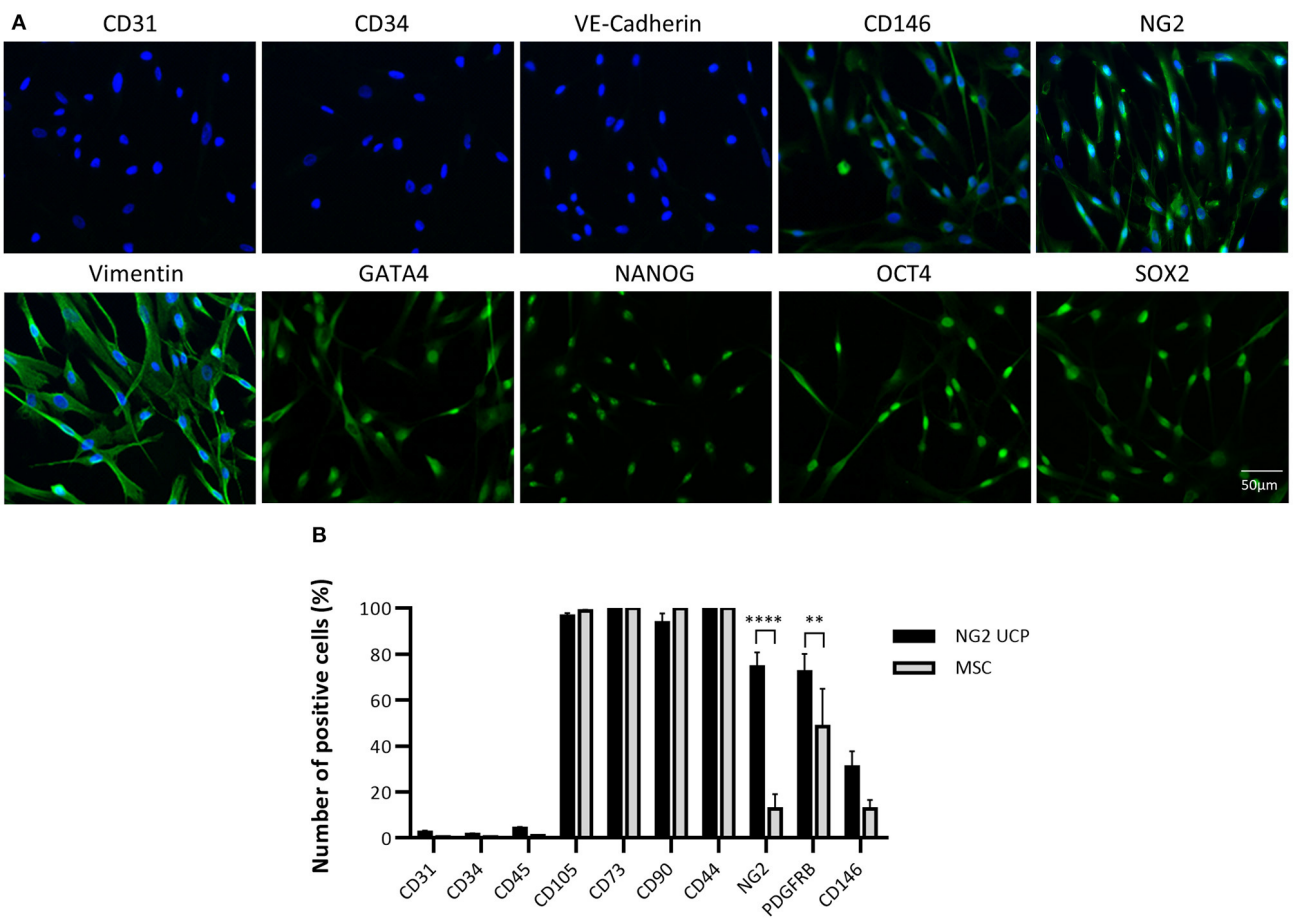
Phenotypic characterization of NG2 UCPs. **(A)** Immunocytochemical staining of NG2 UCPs showing expression of CD146, NG2, vimentin, cardiac transcriptional factor GATA-binding protein 4 (GATA4), transcription factor NANOG, octamer-binding transcription factor 4 (OCT4), and sex-determining region Y-box 2 (SOX2). NG2 UCPs lack expression of endothelial factors CD31, CD34 and vascular endothelial cadherin (VE-Cadherin). **(B)** Summary of the expression profile obtained using flow cytometry. PDGFR-β, platelet-derived growth factor receptor beta. Data represent means (±S.E.M). MSCs and NG2 UCPs, *n* = 5 and 7 biological replicates, respectively. **p < 0.01 and ****p < 0.0001.

To confirm that the isolated cell product represented a pericyte phenotype, an immunocytochemical analysis of NG2 UCPs was completed using an array of antigenic markers (Figure 3A). Cultured cells displayed absence of endothelial cell markers CD31, CD34, and vascular endothelial cadherin (VE-Cadherin) and high expression of pericyte associated markers such as CD146, NG2, and vimentin (8). Furthermore, cells expressed the stemness markers GATA-binding protein 4 (GATA4), homeobox protein NANOG, octamer-binding transcription factor 4 (OCT4) and sex determining region Y-box 2 (SOX2).

To verify that the new isolation protocol produced a consistent homogenous pericyte population, flow cytometry was utilized for further characterization. This confirmed that <5% of immunomagnetic bead sorted NG2 UCP populations expressed endothelial markers CD31 and CD34 or hematopoietic marker CD45 (Figure 3B). Conversely, more than 95% of donor cells displayed mesenchymal markers CD105, CD73, CD90, and CD44, in association with NG2 and platelet derived growth factor receptor beta (PDGFR-β), both >70%. Moreover, only 30% cells were positive for CD146. This antigenic profile was consistent between cell donors and corroborated previous studies which demonstrated *in situ* perivascular expression of mesenchymal markers CD105, CD90, and CD44, but absence of endothelial marker CD34 (21). The profile of NG2 UCPs resembled that of MSCs except for significantly higher expression of NG2 and PDGFR-β in the former. We confirmed that this difference in expression was not due to the effect of culture media as MSCs that were cultured in EGM-2 demonstrated no difference in marker expression to those that were cultured in MSC medium (Supplementary Figure 2).

Together these data indicate that the new isolation protocol provides a consistently homogenous pericytes-like cell product.

### VSMC Differentiation and Contractile Capacity of NG2 UCPs

**Figure 4:**
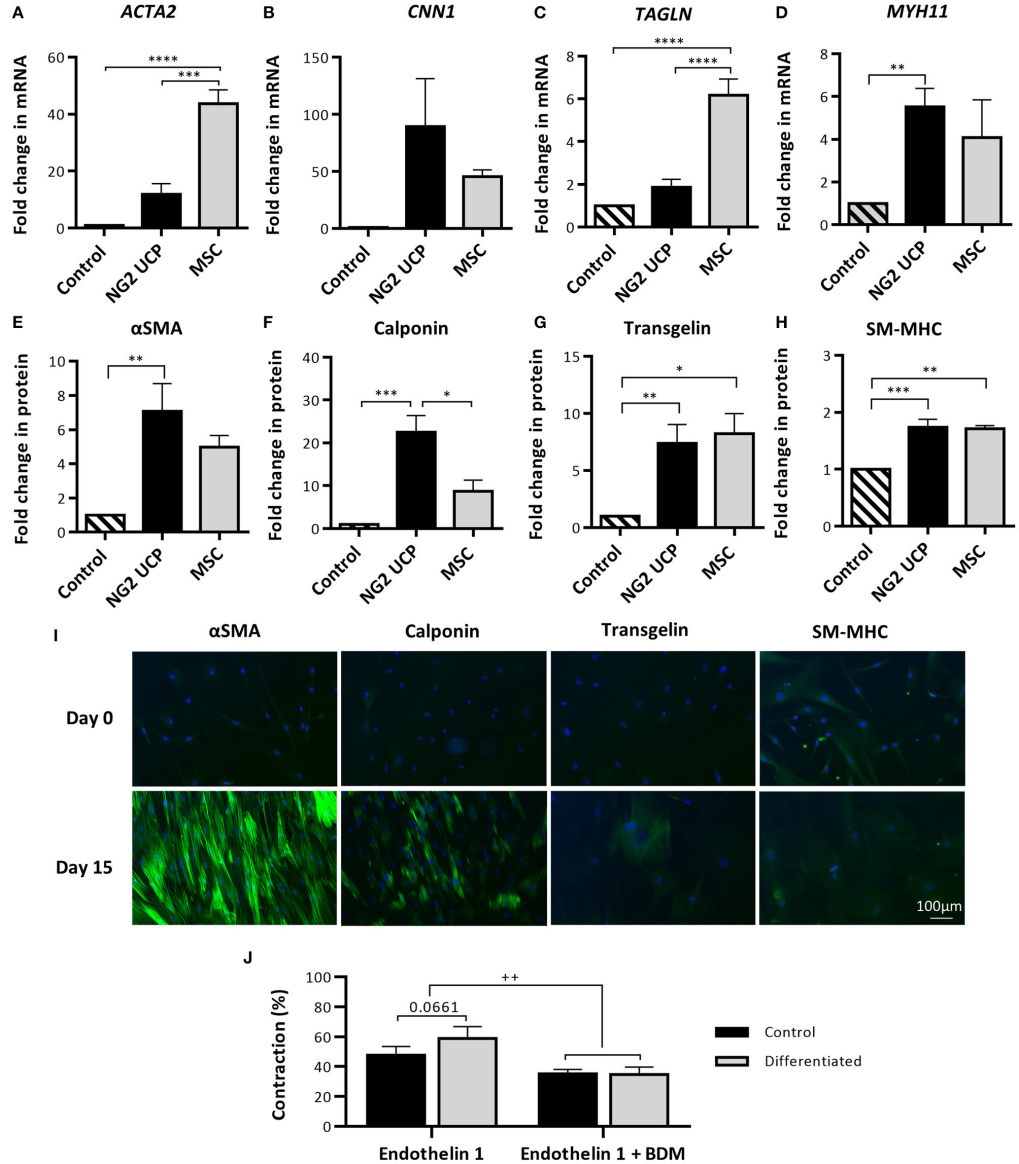
Analysis of NG2 UCP capacity to differentiate toward a contractile vascular smooth muscle-like phenotype. **(A–D)** Fold change in mRNA levels of VSMC markers after differentiation vs. undifferentiated (control). *ACTA2*, αSMA; *CNN1*, Calponin; *TAGLN*, Transgelin; *MYH11*, SM-MHC. **(E–H)** Fold change in protein levels of VSMC markers after differentiation protocol. For **(A–H)**, data represent means (±S.E.M). MSCs and NG2 UCPs, *n* = 3 and 5 biological replicates, respectively; *p < 0.05; **p < 0.01; ***p < 0.001; ****p < 0.0001. **(I)** Immunocytochemical staining of VSMC markers at day 0 (undifferentiated) and day 15 (differentiated). **(J)** Gel contraction in differentiated and undifferentiated NG2 UCPs after activation with endothelin-1 in the presence or absence of BDM. Data represent means (±S.E.M) of *n* = 5 biological replicates. ^++^*p* < 0.01.

To evaluate if NG2 UCPs could form functional smooth muscle-like tissue, cells were exposed to medium containing TGF-β to stimulate differentiation into VSMCs. Differentiated and undifferentiated NG2 UCPs were screened for an array of antigenic markers, which are used to indicate the maturity of the VSMC phenotype (ranging from a synthetic to a mature contractile phenotype). After 15 days exposure to the differentiation medium, NG2 UCP mRNA levels of all antigenic markers were upregulated, although *MYH11* displayed the only significant increase (Figures 4A–D). In comparison, MSCs demonstrated a significantly greater fold change in *ACTA2* and *TAGLN*. Interestingly, at the protein level, differentiated NG2 UCPs demonstrated significant upregulation in all VSMC associated markers, including large increases in αSMA (>6-fold), calponin (>20-fold), and transgelin (>6-fold) (Figures 4E–H). Moreover, detection of SM-MHC at both mRNA and protein levels suggests differentiation toward a specific VSMC phenotype rather than a myofibroblastic phenotype. MSCs demonstrated comparable modulations in αSMA, transgelin and SM-MHC, but less than half the increase in calponin. Figure 4I confirmed the upregulation of VSMC associated markers. No VSMC marker expression was observed in the undifferentiated state, however, differentiated NG2 UCPs acquired the expression of contractile markers αSMA and calponin, along with a modest upregulation in VSMC specific contractile marker transgelin.

The contractile ability of differentiated and undifferentiated NG2 UCPs was assessed to identify if the change in the antigenic phenotype corresponded to functional readouts. After stimulation with endothelin-1, NG2 UCPs showed contraction, more evidently after differentiation (58.9 vs. 47.7% in the undifferentiated state), with this effect being inhibited by BDM, an excitation-uncoupling agent (Figure 4J).

These data indicate that NG2 UCPs have native contractile properties, as seen classically in pericytes, with this property being increased with the acquisition of VSMC antigens during forced differentiation.

### Endothelium Supporting Properties of NG2 UCPs

**Figure 5:**
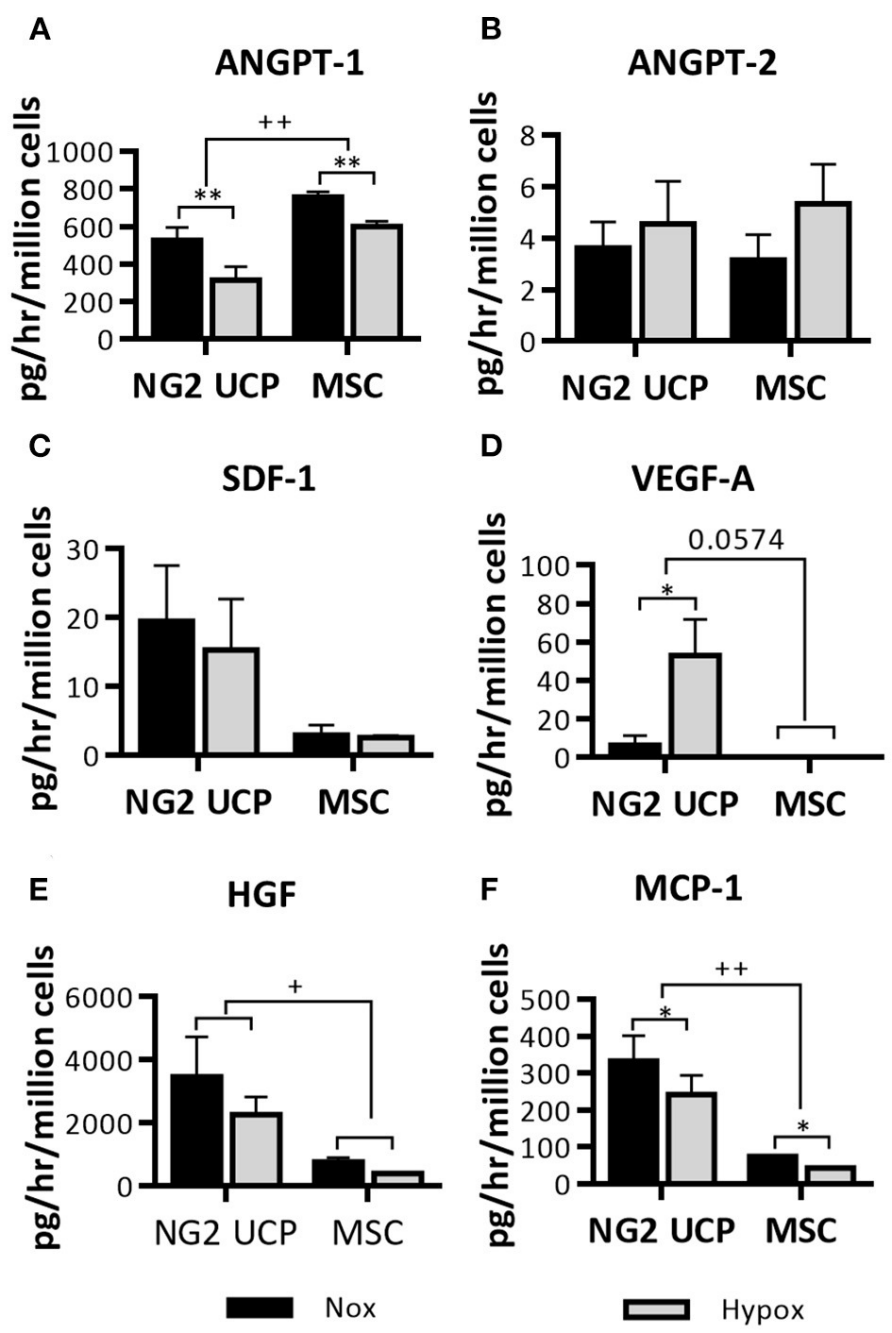
Characterization of the NG2 UCP secretome. **(A–F)** Analysis of cell-secreted factors in cell-conditioned media, normalized for the volume of the collected supernatant, time of incubation and cell number. ANGPT-1, angiopoietin 1; ANGPT-2, angiopoietin; SDF-1, stromal derived factor 1; VEGF-A, vascular endothelial growth factor A; HGF, hepatocyte growth factor; MCP-1, monocyte chemoattractant protein 1. Data represent means (±S.E.M). MSCs *n* = 3, NG2 UCPs *n* = 6; ^*/+^*p* < 0.05 and ^**/++^*p* < 0.01. *represents differences between hypoxia and normoxia, ^+^represents differences between cell types.

Endothelial cell coverage is essential for graft maturation and reduction of thrombotic complications, whilst graft neovascularization should supply the tissue with oxygen and nutrients. NG2 UCPs may facilitate both processes through the release of factors that attract endothelial cells and promote angiogenesis. To test this, the CM collected with cells under normoxic or hypoxic conditions was analyzed for an array of factors (Figures 5A–F). NG2 UCPs secreted different angiocrine factors, some of which showed significant modulation by hypoxia (increase: VEGF-A and reduction: ANGPT-1 and MCP-1). After normalization for the number of cells, NG2 UCPs showed a superior secretory capacity compared with MSCs, except for ANGPT-1, which was higher in MSCs, and ANGPT-2, which was secreted in negligible amounts by both cell types.

**Figure 6:**
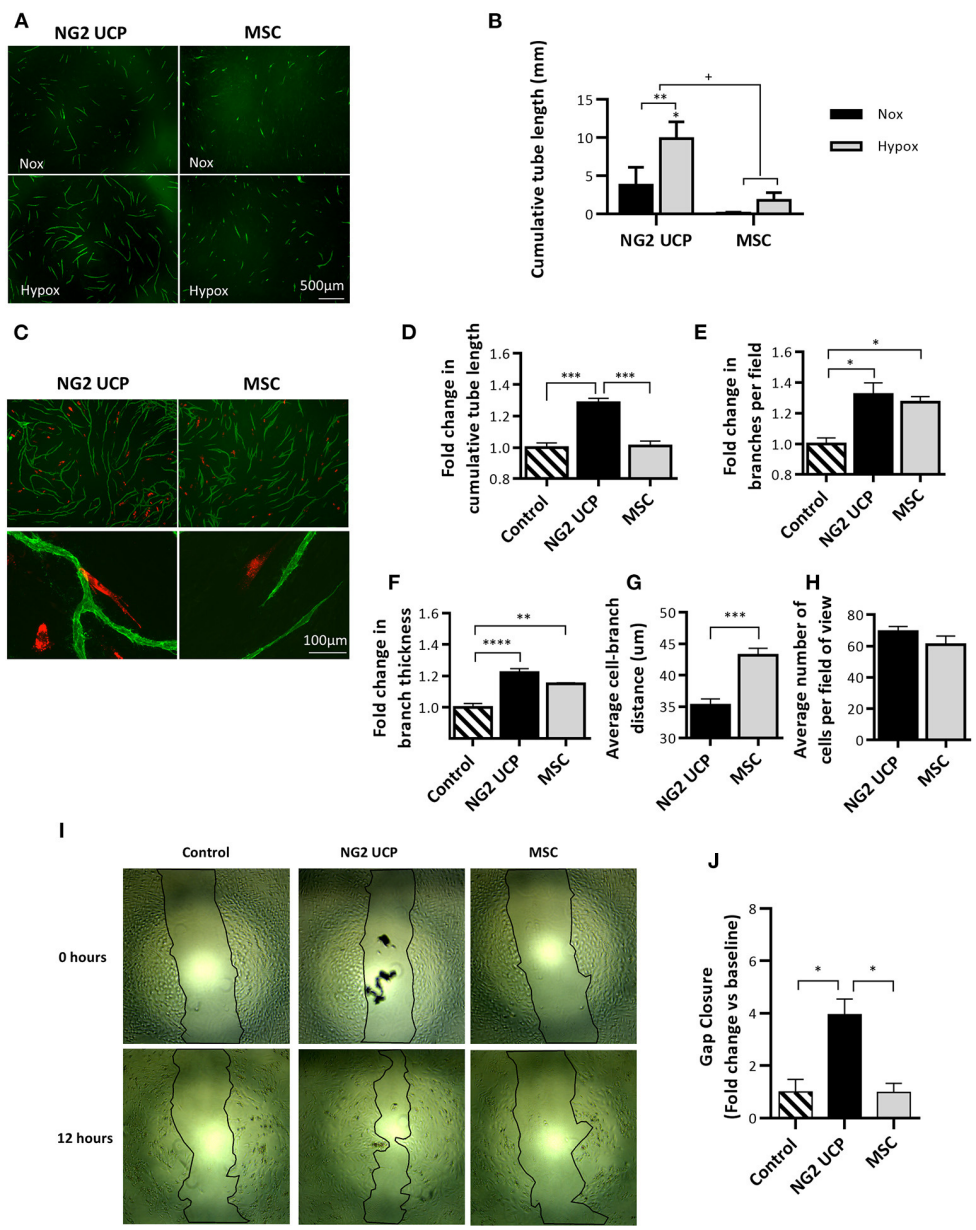
Analysis of NG2 UCP angiogenic potential. **(A)** Representative images of endothelial tube network formation after incubation with normoxic or hypoxic CM. **(B)** Cumulative tube network using normoxic and hypoxic CM. Note that basal medium control resulted in no network formation. For clarity, control has been omitted, however, a significant increase from control is represented directly above NG2 UCP hypoxia column. Data represent means (±S.E.M). MSCs and NG2 UCPs, *n* = 3 and 6 biological replicates, respectively. ^*/+^*p* < 0.05 and **p < 0.01. *represents differences between hypoxia and normoxia, ^+^ represents differences between cell types. **(C)** Representative images of endothelial tube network formation with the inclusion of red labeled NG2 UCPs or MSCs in the endothelial cell-fibroblast co-culture. **(D)** Fold change in cumulative tube length *vs*. endothelial cell-fibroblast only control. **(E)** Fold change in branches per field *vs*. endothelial cell-fibroblast only control. **(F)** Fold change in branch thickness vs. endothelial cell-fibroblast only control. **(G)** Average distance of cell to branch. **(H)** Average number of cells per field of view. For **(D–H)**, data represent means (±S.E.M) MSCs and NG2 UCP, *n* = 4 and 6 biological replicates, respectively. *p < 0.05, **p < 0.01, ***p < 0.001, and ****p < 0.0001. **(I)** Representative scratch-wound migration assay images at 0 and 12 h. **(J)** Summary of fold change in migration *vs*. control. Data represent means (±S.E.M) *n* = 3 biological replicates. *p < 0.05.

The ability of NG2 UCPs to stimulate the formation of endothelial capillary-like networks was assessed using the UCP-derived CM and a 3D co-culture assay. An induction in cumulative tube length was observed using the NG2 UCP-derived CM, particularly the medium collected under hypoxic conditions, which was superior to that of MSC (Figures 6A,B). Next, the direct angiogenic influence was investigated by seeding NG2 UCPs or MSCs together with HUVEC in the co-culture assay (Figure 6C). In accordance with the results from CM, NG2 UCPs significantly increased the tube length in comparison with MSCs (Figure 6D). Both NG2 UCPs and MSCs induced a significant increase in average branches per field and branch thickness (Figures 6E,F). Finally, NG2 UCPs demonstrated a close association with HUVECs as evidenced by their average distance to network branch, which was shorter than that of MSCs (Figure 6G). To confirm the angiogenic effect was not due to increased proliferation of NG2 UCPs, the number of cells per field of view was counted. Although the average number of NG2 UCPs was slightly greater than MSCs (69.2 NG2 UCPs vs. 60.9 MSCs), this difference was not significant (Figure 6H).

Next, using a scratch wound assay, we assessed if NG2 UCPs may accelerate HUVEC migration. We found that ECs incubated with NG2 UCP CM migrated four times farther than HUVECs incubated with either unconditioned medium or MSC CM (Figures 6I,J).

### Ability of NG2 UCPs to Remodel the Extracellular Environment

**Figure 7:**
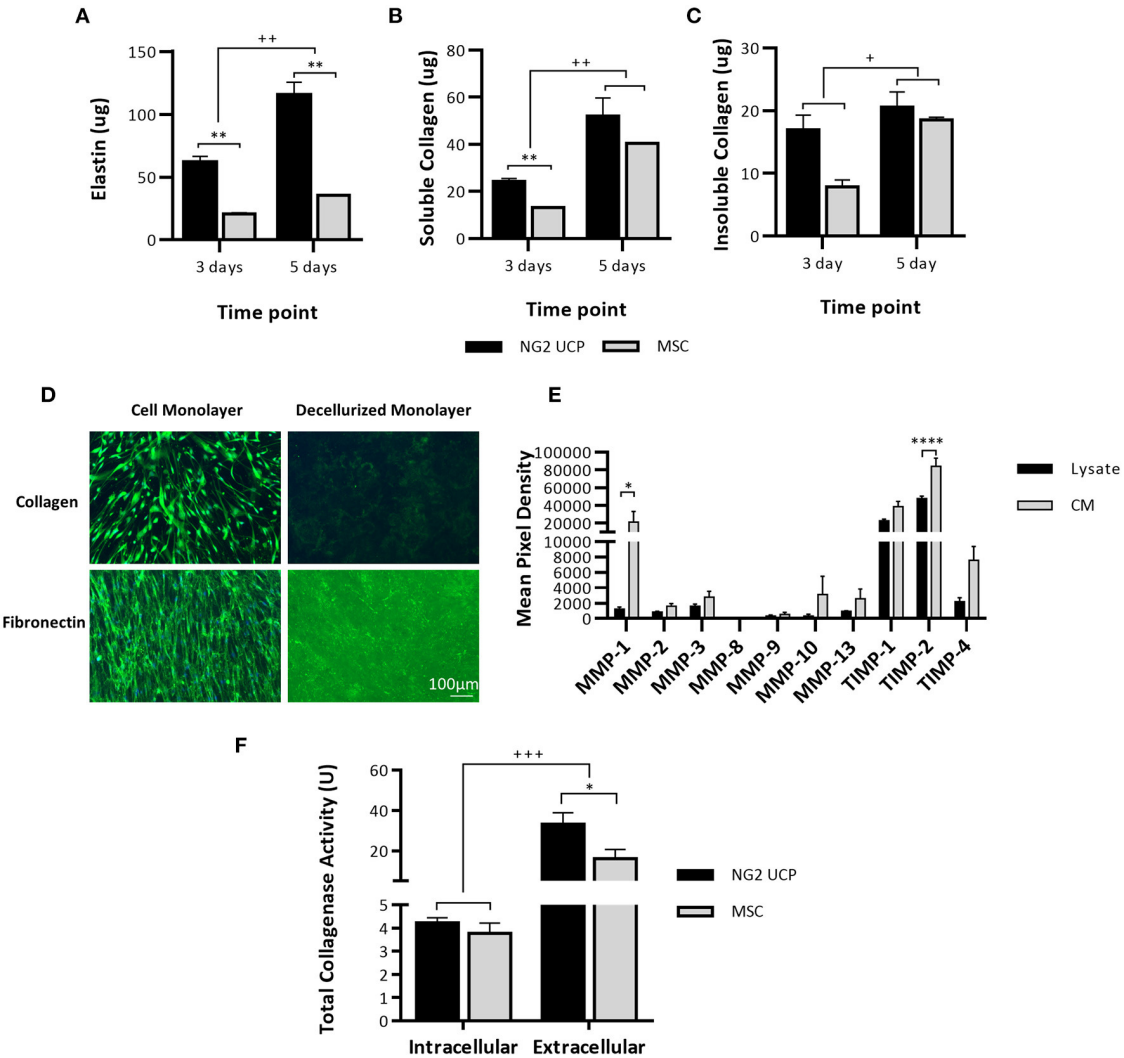
NG2 UCPs produce ECM proteins. **(A)** Quantification of elastin in confluent cell monolayers. **(B)** Quantification of pepsin acid-soluble collagen in confluent cell monolayers and CM. **(C)** Quantification of insoluble collagen in confluent cell monolayers. For **(A–C)**, data represent means (±S.E.M). MSCs and NG2 UCPs, *n* = 2 and 6 biological replicates, respectively. ^*/+^*p* < 0.05 and ^**/++^*p* < 0.01. * represents differences between cell types, ^+^ represents differences between time points. **(D)** Presence of extracellular proteins collagen and fibronectin in decellularized monolayers. **(E)** Relative MMP levels in NG2 UCP lysate and CM. Data represent means (±S.E.M), *n* = 4 biological replicates; *p < 0.05 and ****p < 0.0001. **(F)** Total intracellular (total protein) and extracellular (CM) collagenase activity in NG2 UCPs and MSCs per T25 culture flask. Data represent means (±S.E.M). MSCs and NG2 UCPs, *n* = 4 and 6 biological replicates, respectively. ^*/+^*p* < 0.05 and ^***/+++^*p* < 0.001. * represents differences between cell types, ^+^ represents differences between intracellular and extracellular activity.

We next investigated the capacity of NG2 UCPs to produce ECM proteins and matrix metalloprotease (MMP) when cultured as confluent cell monolayers. Elastin content doubled in UCPs between days 3 and 5, and to a lesser extent in MSCs (Figure 7A). A similar pattern was observed regarding soluble collagen quantified in monolayers and CM. However, the initial superiority of UCPs *vs*. MSCs was lost at 5 days (Figure 7B). Likewise, NG2 UCPs produced more insoluble collagen at 3 days but were similar to MSCs at 5 days (Figure 7C). Immunocytochemical staining for collagen and fibronectin in decellularized monolayers also confirmed NG2 UCPs secrete extracellular proteins (Figure 7D). The presence of MMPs in NG2 UCPs was visualized using an array membrane. In contrast to tissue inhibitors of matrix metalloproteinases (TIMPs), only low levels of MMPs were detected in cell lysates and CM (Figure 7E). The exception to this was MMP-1, which was highly secreted into medium. In line with these results, the extracellular collagenase activity in CM was significantly higher than intracellular activity assessed in the cell lysate, accounting for 97% of the active collagenases (Figure 7F). Furthermore, the extracellular collagenase activity of NG2 UCPs was significantly higher than that of MSCs.

### Generation and Analysis of NG2 UCP Engineered Grafts

**Figure 8:**
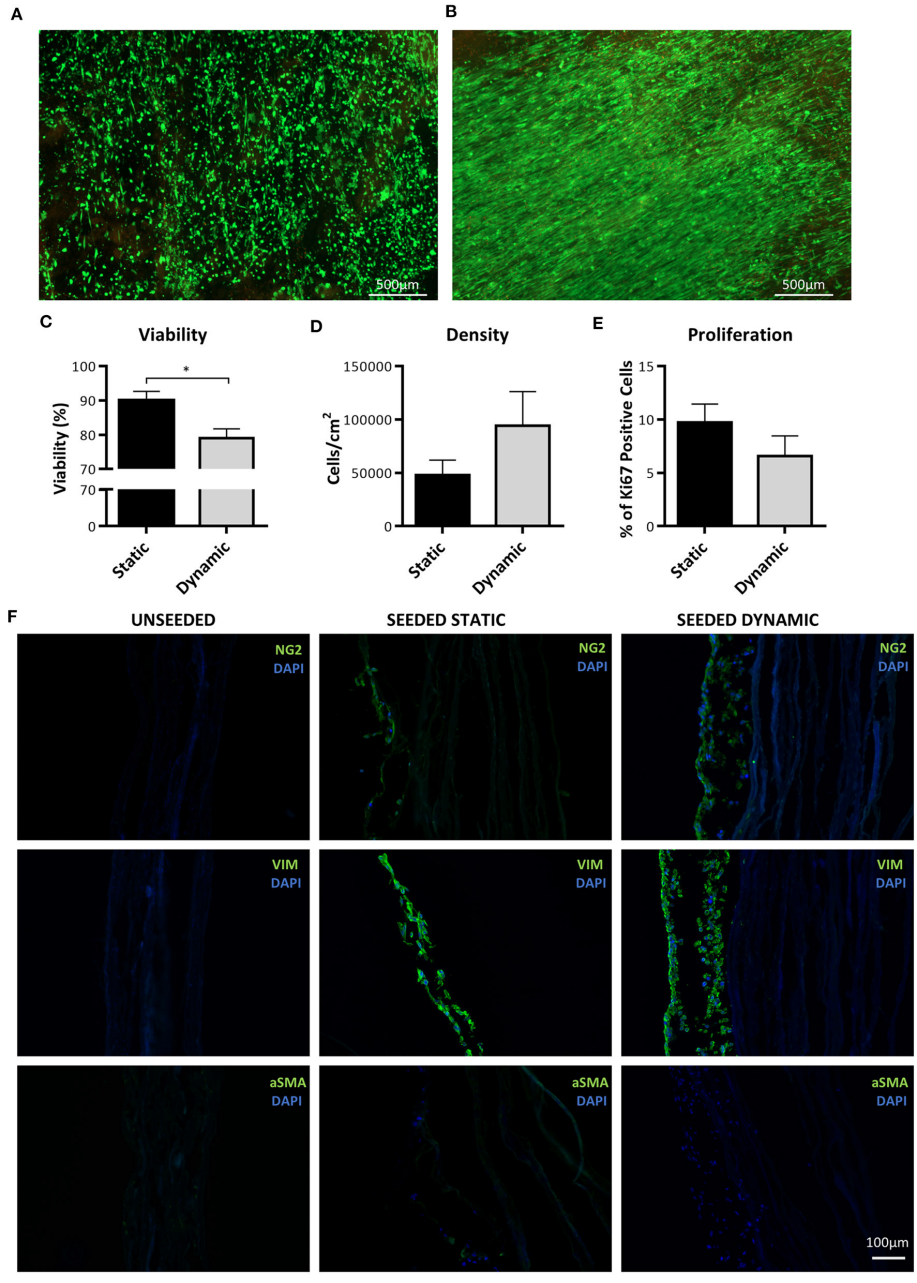
Analysis of cell engraftment on CorMatrix graft. **(A,B)** Representative images of cell distribution and viability after conditioning on CorMatrix scaffold. **(A)** static conditioning. **(B)** dynamic conditioning. Cytoplasmic green fluorescence, live (Calcein AM) cells; Nuclear red fluorescence, dead (ethidium homodimer-1) cells. **(C)** Cell viability. **(D)** Cell density **(E)** Cell proliferation. **(F)** Representative immunohistochemical images demonstrating NG2 UCP presence in graft. Static = 5 days static conditioning, dynamic = 5 days static plus 7 days dynamic conditioning. Data represent means (±S.E.M). *n* = 4 biological replicates. *p < 0.05.

To assess the feasibility of using NG2 UCPs to create a biological graft, cells were seeded on a CorMatrix scaffold at a density of 20,000 cells/cm^2^. The graft was then left in static conditions for 5 days and either analyzed immediately or shaped into a conduit and conditioned for a further 7 days in a flow bioreactor (Supplementary Figure 5). Calcein AM-ethidium homodimer-1 viability staining demonstrated successful attachment of cells to the CorMatrix scaffold (Figures 8A,B). After 7 days dynamic conditioning, the cells were more confluent and aligned in a uniform direction. Cell viability after both static and dynamic conditioning was >75%, however, there was a significant drop in viability after dynamic conditioning (Figure 8C). On average, the cell density increased 2-fold after a 7-day dynamic conditioning, however, the variation between donor cell lines meant this change was not significant (Figure 8D). There was a modest reduction in the number of Ki67 positive cells after dynamic conditioning (Figure 8E). Immunohistochemical analysis of the grafts demonstrated the development of a multi-cell layer on top of the CorMatrix, with cells retaining expression of the pericyte associated markers NG2 and vimentin (Figure 8F). The absence of αSMA was interpreted as the lack of spontaneous differentiation of engrafted cells toward a contractile phenotype. As expected, the control unseeded grafts had no cells nor NG2, Vimentin or αSMA expression.

**Figure 9:**
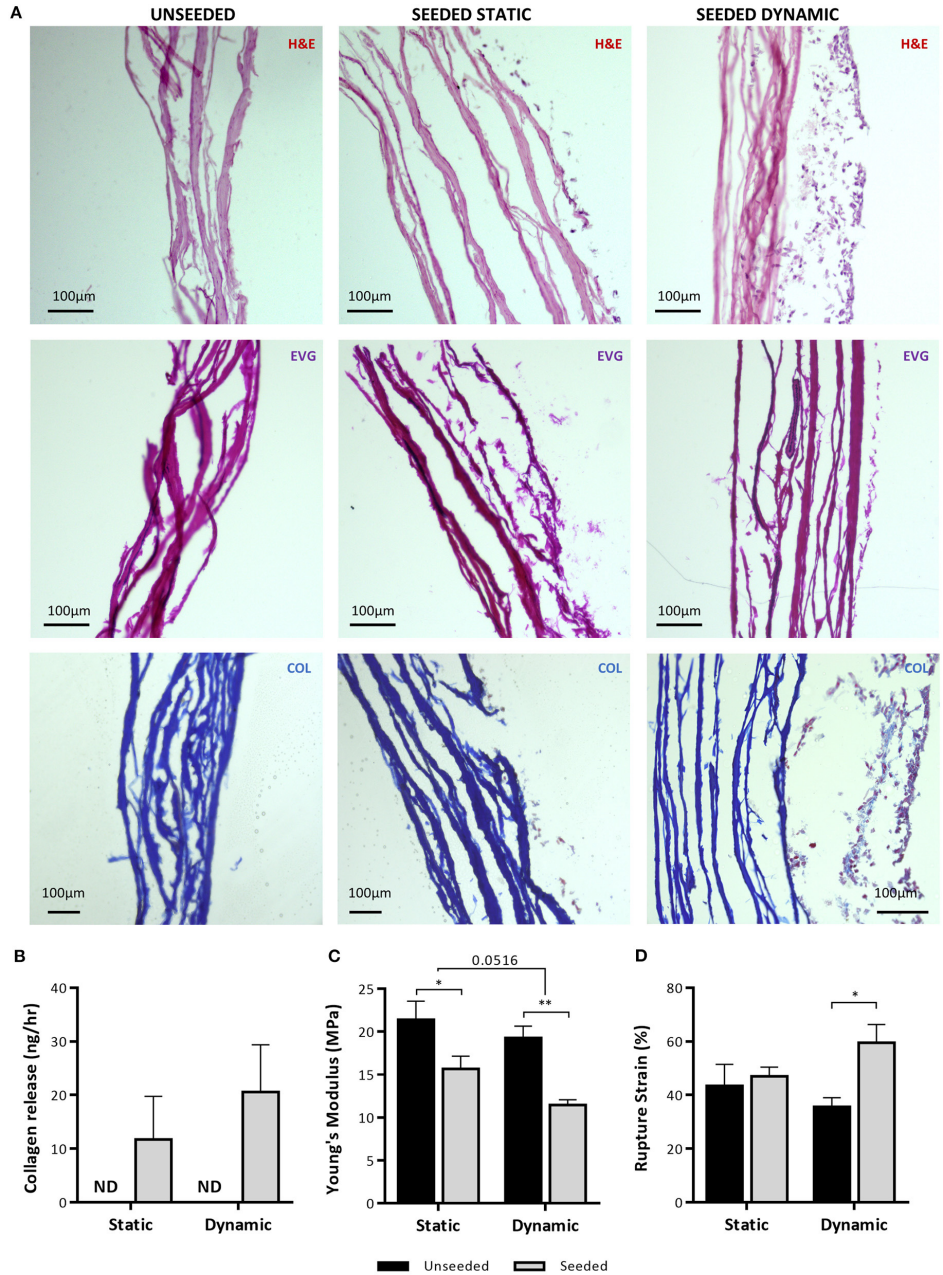
Analysis of NG2 UCP engineered graft structure and properties. **(A)** Representative images of hematoxylin and eosin staining (H&E), elastin van Gieson staining (EVG) and Mallory's trichrome collagen staining (COL) of NG2 UCP seeded CorMatrix grafts. **(B)** Quantification of Pro-Collagen 1 a1 in CM. **(C)** Quantification of Young's Modulus. **(D)** Quantification of rupture strain. Static = 5 days static conditioning, dynamic = 5 days static plus 7 days dynamic conditioning. Data represent means (±S.E.M). Unseeded *n* = 3, and seeded *n* = 4 biological replicates. *p < 0.05 and **p < 0.01.

Finally, H&E staining demonstrated the development of a cellular layer in the UCP-seeded grafts, which appeared to mature and thicken after dynamic conditioning (Figure 9A). Although no new elastin fibers were detected, collagen deposits were identified within the cellular layer after dynamic conditioning, as highlighted by the light blue stain. Analysis of CM collected after static and dynamic conditioning identified the presence of soluble collagen, which was not detected in unseeded grafts (Figure 9B). Importantly, we could appreciate a significant reduction in the Young's Modulus of UCP-seeded grafts in comparison to the unseeded control, indicating an increase in elasticity (Figure 9C). Dynamic conditioning appeared to further increase elasticity. In concurrence with this, there was a significant increase in rupture strain in seeded grafts, which was particularly evident after dynamic conditioning (Figure 9D).

Altogether, these results highlight the ability of UCPs to engraft and proliferate within the CorMatrix scaffold, changing the ECM protein content toward an elastic phenotype.

## Discussion

Our previous work provided a rationale for the use of human pericytes from the neonatal heart to engineer clinically available prosthetic grafts (9, 14). However, isolation of this promising cell population is reliant upon tissue obtained during initial palliative surgery or an *ad hoc* invasive cardiac biopsy. In search of a more accessible source of pericytes, we focused on the umbilical cord, which is acknowledged to contain stromal cells and perivascular cells endowed with regenerative capacity. We developed a standard protocol for pericyte isolation, based on immunomagnetic separation of NG2 positive cells from the explant outgrowth produced by umbilical arteries. Having confirmed that NG2 positive cells share typical antigenic properties, perivascular location, and paracrine activities indicative of pericytes, we demonstrated their ability to promote HUVEC migration and tube formation. In functional assays, NG2 UPCs outperformed Wharton's Jelly-derived MSCs. Finally, we demonstrated that NG2 UPCs can engraft and grow onto a CorMatrix graft, changing its mechanical properties toward an elastic behavior.

The umbilical cord starts to form at about 4 weeks of pregnancy and usually grows to about 22 inches long at birth. Previous studies using umbilical artery dissection and culture selection identified this tissue as a source of perivascular mesenchymal stromal cells suitable for regenerative medicine applications. However, only cells that were collected during early gestation expressed stemness markers and exhibited a high proliferative potential and multilineage differentiation capacity compared with full-term counterparts, suggesting a restriction in regenerative activity of these cells occurs through gestation (22). Alternatively, the method used may be not adequate to separate and expand the small number of progenitors contained within the bulk cell population of a full-term cord artery. We employed immunomagnetic sorting to separate NG2 positive cells from colonies outgrowing from the umbilical artery of full-term neonates, after initial unsuccessful attempts based on CD34 immunomagnetic selection (14, 15). CD34 is a transmembrane glycoprotein protein typically expressed by adventitial pericytes; whereas umbilical vessels do not possess a distinct adventitia (23). Indeed, Schugar et al. previously demonstrated that CD34 expression may be restricted to the endothelial layer in the umbilical vessels (21). Accordingly, *in situ* immunohistochemistry confirmed the absence of CD31^−^/CD34^+^ pericyte-like cells in umbilical vessels. Surprisingly, we only identified sporadic staining of NG2 positive cells within the tissue, whereas Crisan et al. identified a distinct NG2 positive pericyte layer within the umbilical artery (24). This result may be due to different methods of tissue processing. Nevertheless, the study by Crisan et al. indicates that NG2 is a suitable selection marker for umbilical cord pericytes.

Antigenic characterization confirmed that expanded NG2 positive cells resemble *bona fide* pericytes, therefore we coined for them the term of NG2 UCPs. Phenotypically, NG2 UCPs are similar to cardiac and saphenous vein pericytes, except they moderately express CD146, which is typically not found in pericytes isolated using CD31^−^/CD34^+^ selection (14, 15). Similarly, they also express stemness markers, rapidly proliferate in culture to 10 million cells in under 6 weeks and can be induced to a contractile VSMC phenotype, although we did not detect acquisition of a mature phenotype, as seen in cardiac pericytes. Nevertheless, this data suggests that a subfraction of umbilical cord perivascular cells maintain a progenitor cell phenotype through gestation.

By analyzing secreted growth factors we aimed to understand the NG2 UPC angiogenic potential, and how this may change in response to an oxygen poor environment, such as an avascular tissue engineered construct (25, 26). In fact, engineered tissues thicker than 1 mm are prone to become necrotic before perfusion by host-derived microvessels (27, 28). A cell that can adapt to this environment effectively may be favorable for tissue engineering. We compared NG2 UCPs to a commercial line of Wharton's Jelly MSCs, which are often the focus of cell therapy and tissue engineering applications (29, 30). NG2 UCPs remarkably outperformed their comparator regarding the ability to release SDF-1, VEGF-A, HGF, and MCP-1 under normoxia and hypoxia, while showing moderately lower levels of ANGPT-1. Interestingly, this differed quite considerably from cardiac pericytes, which secreted significantly more VEGF-A, HGF and ANGPT-2 (14). Using a fibroblast-endothelial cell coculture assay, we confirmed the superior proangiogenic potential of NG2 UCPs, as highlighted by greater endothelial network formation induced by their CM and closer contacts with network branches in comparison with MSCs. This result was comparable to tube formation data from cardiac pericytes; however, intriguingly the angiogenic paracrine effect of NG2 UCPs appears superior (14). This may be due to the high secretion of ANGPT-2 seen in cardiac pericytes, which in the absence of angiogenic stimulation results in vessel regression (31). Additionally, data indicates that NG2 UCPs may help facilitate the development of a graft endothelial layer. In animal models of transplanted vascular grafts, reendothelialization tends to occur through trans-anastomotic ingrowth, however, in humans this process is much slower and limited to <2 cm (32, 33). Consequently, this leads to incomplete endothelial layer formation and thrombotic complications. Through implementation of a scratch wound migration model we showed that CM from NG2 UCPs enhanced EC migration, which may increase efficiency of trans-anastomotic ingrowth. Furthermore, the proangiogenic properties of NG2 UCPs may support transmural capillarization, which has been identified as a more feasible mechanism of reendothelialization in humans (32). Conversely, saphenous vein pericyte CM do not influence endothelial migration (15). Together, this data suggests NG2 UCPs may possess superior paracrine endothelial supportive properties than pericyte populations previously isolated by our group.

The ECM of native arteries can dynamically remodel through a tightly regulated balance of matricellular protein production and MMP activity, whereas this dynamic phenomenon is lost in acellular grafts, resulting in lack of growth potential and susceptibility to degradation (34). The main constituents of vascular ECM are collagens and elastin, which are present in different proportions according to the type and anatomical location of the tissue. In fact, the arterial wall is made up of up to 50% elastin, which provides mechanical integrity and vessel elasticity (35). Conversely, CorMatrix is made up of 90% collagen, predominantly collagen type I (36). A lack of elastin synthesis in tissue engineered vascular grafts has been highlighted as a major limitation to successful outcomes (37). Noteworthy, we have shown that NG2 UCPs produce significant amounts of both collagen and elastin, which suggests that seeding of these cells may improve mechanical properties of acellular scaffolds. Naturally, this ECM production must be regulated to ensure there is minimal risk of pathological remodeling. In accordance with this, we analyzed MMP presence and activity in isolated cells. Although general intracellular MMP levels were low in comparison to TIMPs, we saw significantly increased levels of extracellular MMP-1. This observation was mirrored by an increased extracellular collagenase activity, suggesting MMPs may become activated upon secretion. MMP-1 acts on collagen type I, the predominant collagen in animal derived ECM such as CorMatrix, suggesting that NG2 UCPs could degrade and remodel this material (38).

Finally, we explored the feasibility of engineering a biological graft by incorporating NG2 UCPs onto CorMatrix. Analysis of cell distribution demonstrated a largely uniform engraftment, although some areas where the conduit had been gripped were sparsely populated and had an increased number of apoptotic cells. Specifically, there was a drop in viability after dynamic conditioning. Improvement in physical manipulation of the scaffold is warranted to maintain cell viability from static to dynamic conditions. Interestingly, NG2 UCPs demonstrated organization after stimulation under flow conditions, which may have important implications for the mechanical properties of the graft (39). Immunohistochemical staining demonstrated that NG2 UCPs retain their original phenotype, with the absence of αSMA suggesting that additional stimuli and/or prolonged shear stress are required to encourage development of a smooth muscle layer. Analysis also revealed that the cells form a layer on the external surface of the CorMatrix scaffold rather than infiltrate into the matrix. Whilst this lack of infiltration initially looked concerning, it is not necessarily a poor outcome. The purpose of cellularizing the exterior surface of the CorMatrix conduit was to create an adventitial cellular layer capable of recruiting native vascular cells and repopulating the acellular scaffold through the therapeutic properties of NG2 UCPs.

No new elastin fibers were detected in the cellularized graft; however, it is possible that 12 days were not enough for fiber development. Nonetheless, evidence of scaffold remodeling was detected through progressive collagen deposition and the presence of soluble collagen (alpha-1 type I collagen) in the conditioned medium. Alpha-1 type I collagen is a major component of collagen type 1; its presence may indicate that the cells are actively remodeling the collagen in the CorMatrix scaffold. In accordance with this, we observed a considerable change in mechanical properties of the graft. Although the cellularized graft still remained stiffer than native arterial tissue, the reduction in Young's modulus demonstrated a significant increase in elasticity from the acellular graft (40). Furthermore, the increase in rupture strain suggests an increased compliance of the graft, which is essential for normal arterial function (41). These data demonstrate that the NG2 UCP-engineered graft possesses significant improvements over acellular CorMatrix, which is an FDA-approved material widely used in cardiovascular reconstructive surgery of pediatric and adult patients.

## Conclusion

We have demonstrated that a novel population of NG2^+^ pericytes can be consistently isolated and expanded from discarded umbilical tissue. The acquired cell population demonstrates high reproducibility and a homogenous phenotype. Furthermore, NG2^+^ pericytes possess specific properties that make them a desirable candidate for vascular tissue engineering in patients with CHD. The ability of NG2 UCPs to produce their own ECM could pave the way to the implementation of clinically available grafts but also to the manufacture of novel grafts made of umbilical vascular cells and a native stromal matrix.

## Data Availability Statement

The raw data supporting the conclusions of this article will be made available by the authors, without undue reservation.

## Author Contributions

WC: conception, design, collection, assembly of data, data analysis, interpretation, and manuscript writing. AF and EJ: collection, assembly of data, data analysis, and interpretation. IR-A and ACT: collection and assembly of data. EA and MC: conception and design. PM: grant holder, conception, design, data analysis, and interpretation. All authors read and approved the final manuscript.

## Conflict of Interest

The authors declare that the research was conducted in the absence of any commercial or financial relationships that could be construed as a potential conflict of interest.
