# Supplementary material for: Umbilical Cord Pericytes Provide a Viable Alternative to Mesenchymal Stem Cells for Neonatal Vascular Engineering
Source: Front Cardiovasc Med. 2021 Jan 21;7:609980. doi: 10.3389/fcvm.2020.609980 (PMC7859275; doi:10.3389/fcvm.2020.609980)
Supplement: Supplementary file 1 [file Table_1.docx]

**Supplementary table 1: Primary antibodies used for immunocytochemistry, immunohistochemistry and western blotting**

| **Antibody** | **Dilution** | **Host Species** | **Supplier** |
| --- | --- | --- | --- |
| aSMA | 1:100 (ICC, IHC) 1:5000 (WB) | Mouse | DAKO (M0851) |
| B-Tubulin | 1:5000 (WB) | Mouse | Cell Signalling Technology |
| Calponin | 1:100 (ICC), 1:5000 (WB) | Rabbit | Abcam (ab46794) |
| CD31 | 1:50 (ICC), 1:100 (IHC) | Mouse | R&D systems (BBA7) |
| CD34 | 1:50 (ICC) | Mouse | DAKO (7165) |
| CD34 | 1:100 (IHC) | Sheep | R&D systems (AF7227) |
| CD146 | 1:100 (ICC) | Rabbit | Abcam (ab75769) |
| Collagen I | 1:100 (ICC) | Rabbit | Chemicon (AB745) |
| Fibronectin | 1:200 (ICC) | Rabbit | Abcam (ab299) |
| GATA-4 | 1:100 (ICC) | Rabbit | Abcam (ab61767) |
| NANOG | 1:100 (ICC) | Rabbit | Abcam (ab80892) |
| NG2 | 1:50 (ICC, IHC) | Rabbit | Millipore (AB-5320) |
| OCT-4 | 1:100 (ICC) | Rabbit | Abcam (ab18976) |
| SM-MHC | 1:100 (ICC), 1:500 (WB) | Rabbit | Abcam (ab53219) |
| SOX2 | 1:100 (ICC) | Rabbit | Millipore (AB5603) |
| Transgelin | 1:50 (ICC), 1:500 (WB) | Mouse | Santa Cruz (sc-271719) |
| VE-Cadherin | 1:50 (ICC) | Mouse | Santa Cruz (SC-9989) |
| Vimentin | 1:400 (ICC, IHC) | Rabbit | Abcam (ab92547) |

ICC, immunocytochemistry; IHC, immunohistochemsitry; WB, western blot
