# Supplementary material for: Umbilical Cord Pericytes Provide a Viable Alternative to Mesenchymal Stem Cells for Neonatal Vascular Engineering
Source: Front Cardiovasc Med. 2021 Jan 21;7:609980. doi: 10.3389/fcvm.2020.609980 (PMC7859275; doi:10.3389/fcvm.2020.609980)
Supplement: Supplementary file 2 [file Table_2.docx]

**Supplementary table 2: Secondary antibodies used for immunocytochemistry and western blotting**

| **Antibody** | **Dilution** | **Supplier** |
| --- | --- | --- |
| Donkey-anti-Sheep Alexa-488 | 1:200 (IHC) | Life Technologies |
| Goat-anti-Mouse Alexa-488 | 1:200 (ICC, IHC) | Life Technologies |
| Goat-anti-Rabbit Alexa-488 | 1:200 (ICC, IHC) | Life Technologies |
| Goat-anti-Mouse Alexa-568 | 1:200 (IHC) | Life Technologies |
| Goat-anti-Rabbit Alexa-647 | 1:200 (IHC) | Life Technologies |
| Donkey-anti- Mouse ECL-HRP | 1:10000 (WB) | GE Healthcare |
| Donkey-anti- Rabbit ECL-HRP | 1:10000 (WB) | GE Healthcare |

ICC, immunocytochemistry; IHC, immunohistochemistry; WB, western blot
