# Supplementary material for: Umbilical Cord Pericytes Provide a Viable Alternative to Mesenchymal Stem Cells for Neonatal Vascular Engineering
Source: Front Cardiovasc Med. 2021 Jan 21;7:609980. doi: 10.3389/fcvm.2020.609980 (PMC7859275; doi:10.3389/fcvm.2020.609980)
Supplement: Supplementary file 3 [file Table_3.docx]

**Supplementary table 3: Antibodies used in flow cytometry studies**

| **Antibody** | **Fluorophore** | **Dilution** | **Supplier** |
| --- | --- | --- | --- |
| CD31 | FITC | 1:10 | BD |
| CD34 | FITC | 1:20 | Miltenyi |
| CD44 | APC | 1:500 | eBioscience |
| CD45 | PerCP-Cy5.5 | 2:25 | Miltenyi |
| CD73 | PE-Cy7 | 3:100 | BioLegend |
| CD90 | PE-Cy7 | 3:100 | BioLegend |
| CD105 | APC | 1:25 | Life Tech |
| CD146 | PerCP-Cy5.5 | 1:10 | R&D |
| NG2 | PE | 1:20 | BD |
| PDGFR-B | PE | 3:100 | BioLegend |
