# Supplementary material for: Umbilical Cord Pericytes Provide a Viable Alternative to Mesenchymal Stem Cells for Neonatal Vascular Engineering
Source: Front Cardiovasc Med. 2021 Jan 21;7:609980. doi: 10.3389/fcvm.2020.609980 (PMC7859275; doi:10.3389/fcvm.2020.609980)
Supplement: Supplementary file 4 [file Table_4.docx]

**Supplementary table 4: Taqman^TM^ probes used for RT-qPCR**

| **Target Gene** | **Probe ID** |
| --- | --- |
| *ACTA2* (aSMA) | HS00426835 |
| *CNN1* (Calponin) | HS00154543 |
| *MYH11* (SM-MHC) | HS00975786 |
| *TAGLN* (Transgelin) | HS01038777 |
| *UBC* (Ubiquitin C; Housekeeper) | HS00824723 |
