# Supplementary material for: Umbilical Cord Pericytes Provide a Viable Alternative to Mesenchymal Stem Cells for Neonatal Vascular Engineering
Source: Front Cardiovasc Med. 2021 Jan 21;7:609980. doi: 10.3389/fcvm.2020.609980 (PMC7859275; doi:10.3389/fcvm.2020.609980)
Supplement: Supplementary file 5 [file Presentation_1.PPTX]

## Slide 1
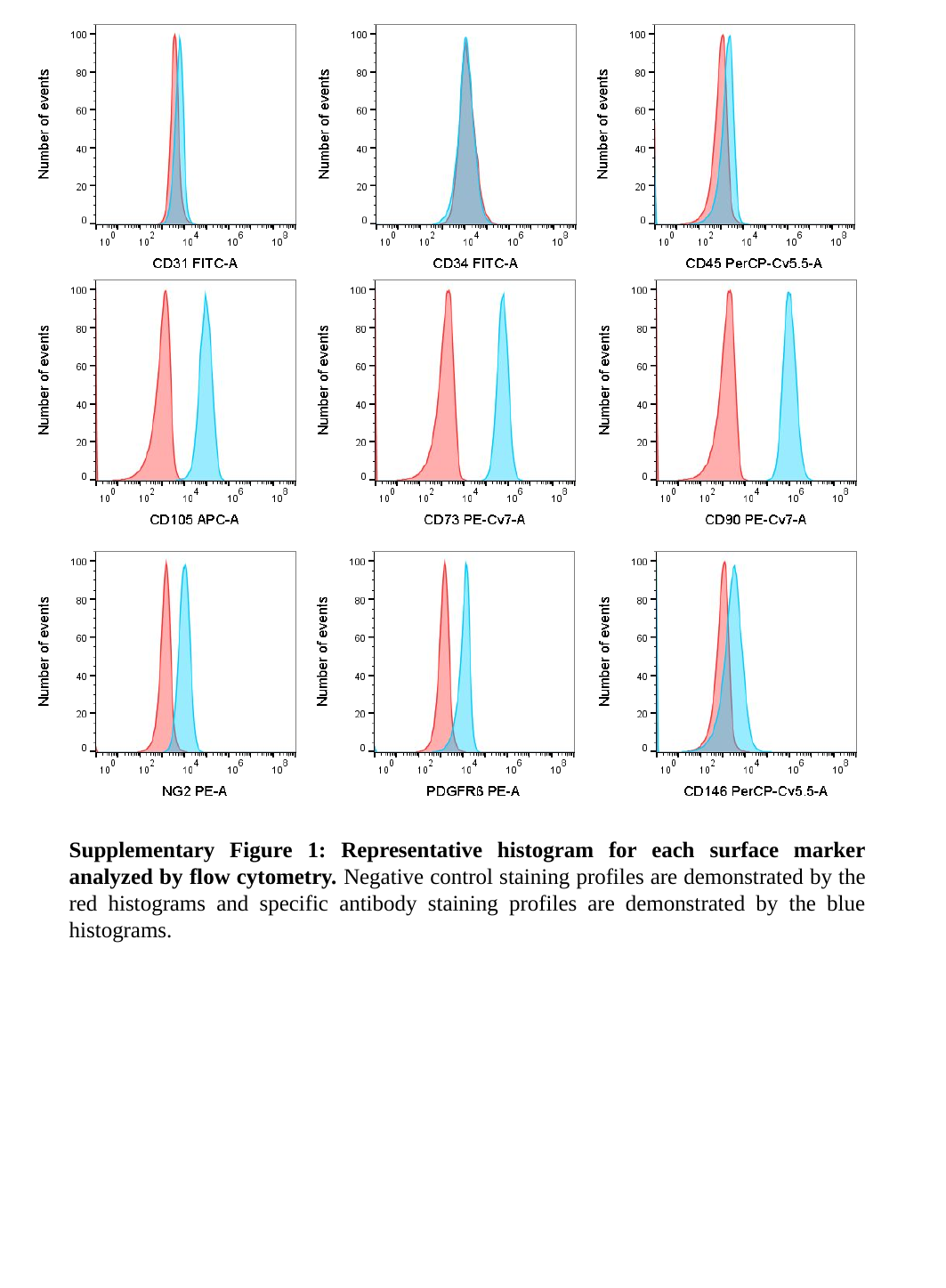

Supplementary Figure 1: Representative histogram for each surface marker analyzed by flow cytometry. Negative control staining profiles are demonstrated by the red histograms and specific antibody staining profiles are demonstrated by the blue histograms.

## Slide 2
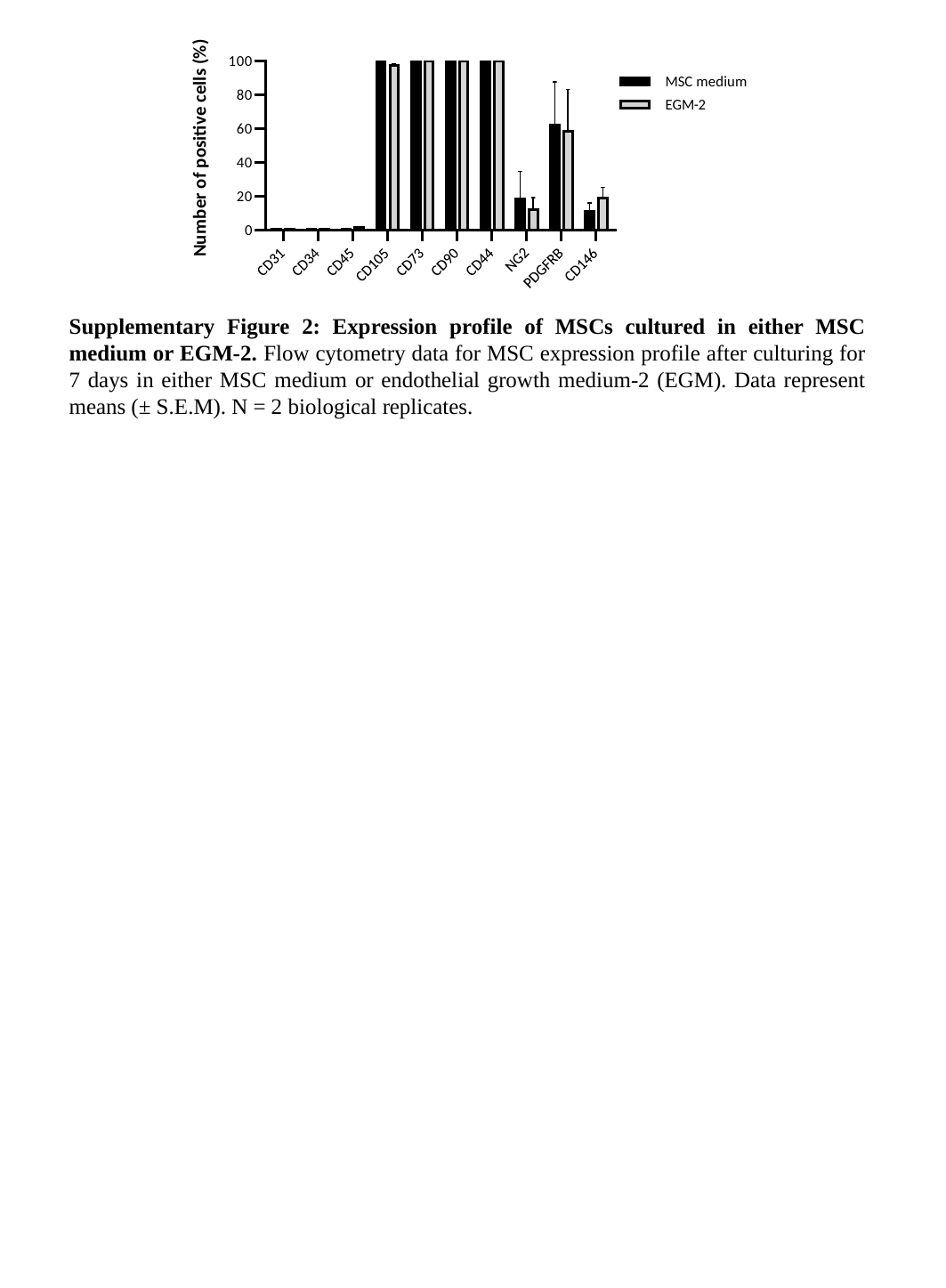

Supplementary Figure 2: Expression profile of MSCs cultured in either MSC medium or EGM-2. Flow cytometry data for MSC expression profile after culturing for 7 days in either MSC medium or endothelial growth medium-2 (EGM). Data represent means (± S.E.M). N = 2 biological replicates.

## Slide 3
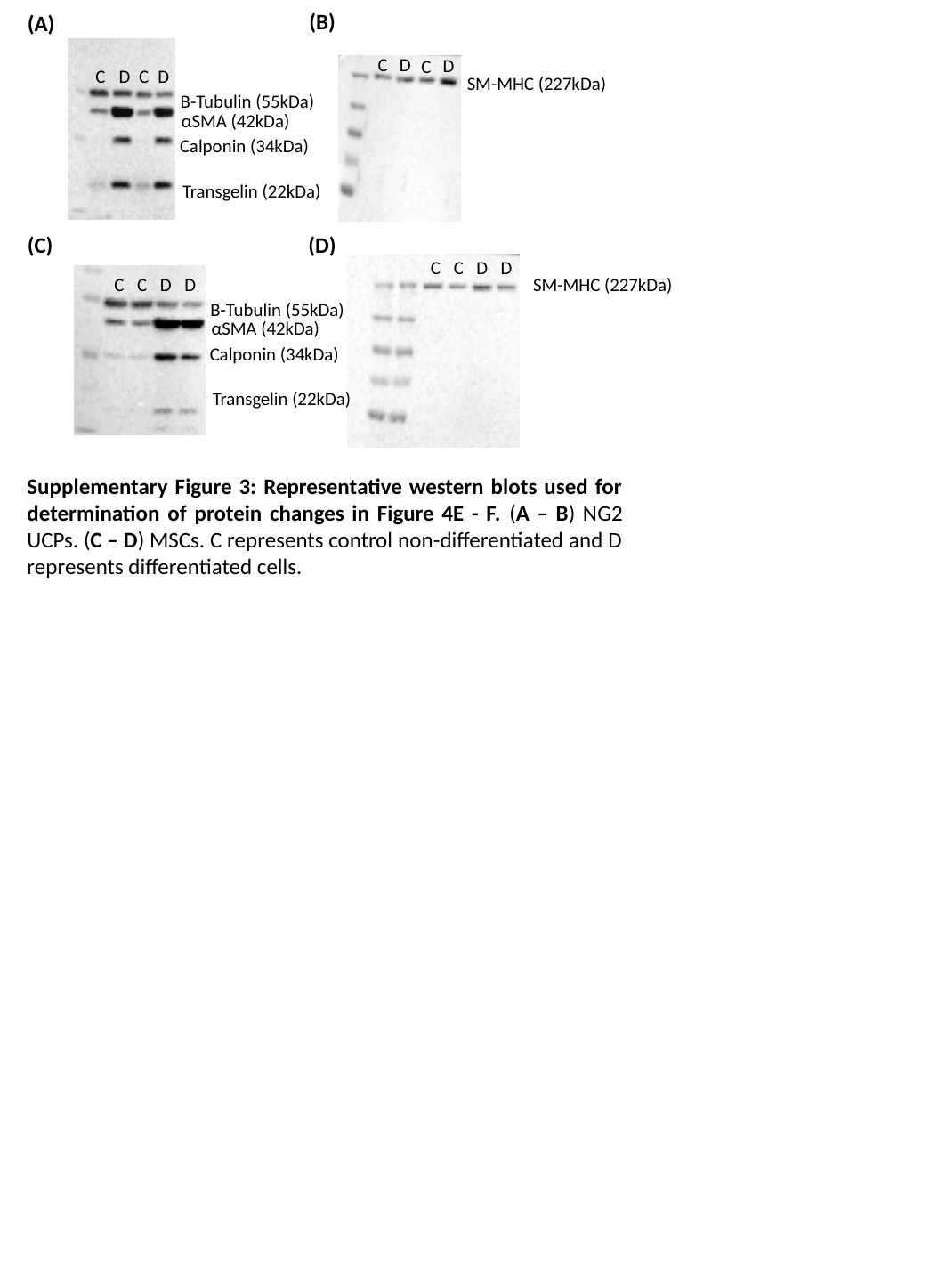

(B)
(A)
C
D
D
C
C
D
C
D
SM-MHC (227kDa)
Β-Tubulin (55kDa)
αSMA (42kDa)
Calponin (34kDa)
Transgelin (22kDa)
(D)
(C)
C
C
D
D
SM-MHC (227kDa)
C
C
D
D
Β-Tubulin (55kDa)
αSMA (42kDa)
Calponin (34kDa)
Transgelin (22kDa)
Supplementary Figure 3: Representative western blots used for determination of protein changes in Figure 4E - F. (A – B) NG2 UCPs. (C – D) MSCs. C represents control non-differentiated and D represents differentiated cells.

## Slide 4
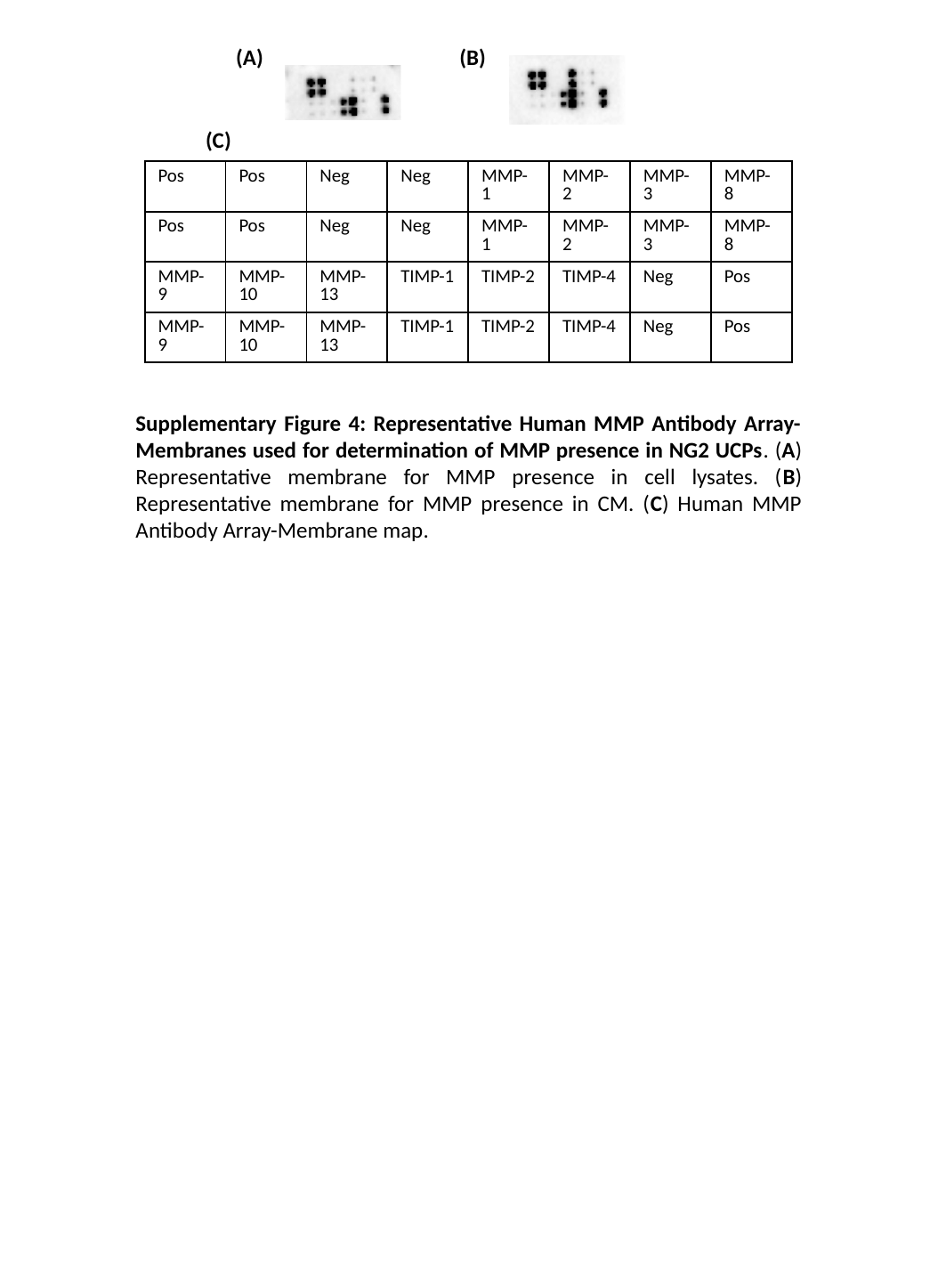

(A)
(B)
(C)
| Pos | Pos | Neg | Neg | MMP-1 | MMP-2 | MMP-3 | MMP-8 |
| --- | --- | --- | --- | --- | --- | --- | --- |
| Pos | Pos | Neg | Neg | MMP-1 | MMP-2 | MMP-3 | MMP-8 |
| MMP-9 | MMP-10 | MMP-13 | TIMP-1 | TIMP-2 | TIMP-4 | Neg | Pos |
| MMP-9 | MMP-10 | MMP-13 | TIMP-1 | TIMP-2 | TIMP-4 | Neg | Pos |
Supplementary Figure 4: Representative Human MMP Antibody Array-Membranes used for determination of MMP presence in NG2 UCPs. (A) Representative membrane for MMP presence in cell lysates. (B) Representative membrane for MMP presence in CM. (C) Human MMP Antibody Array-Membrane map.

## Slide 5
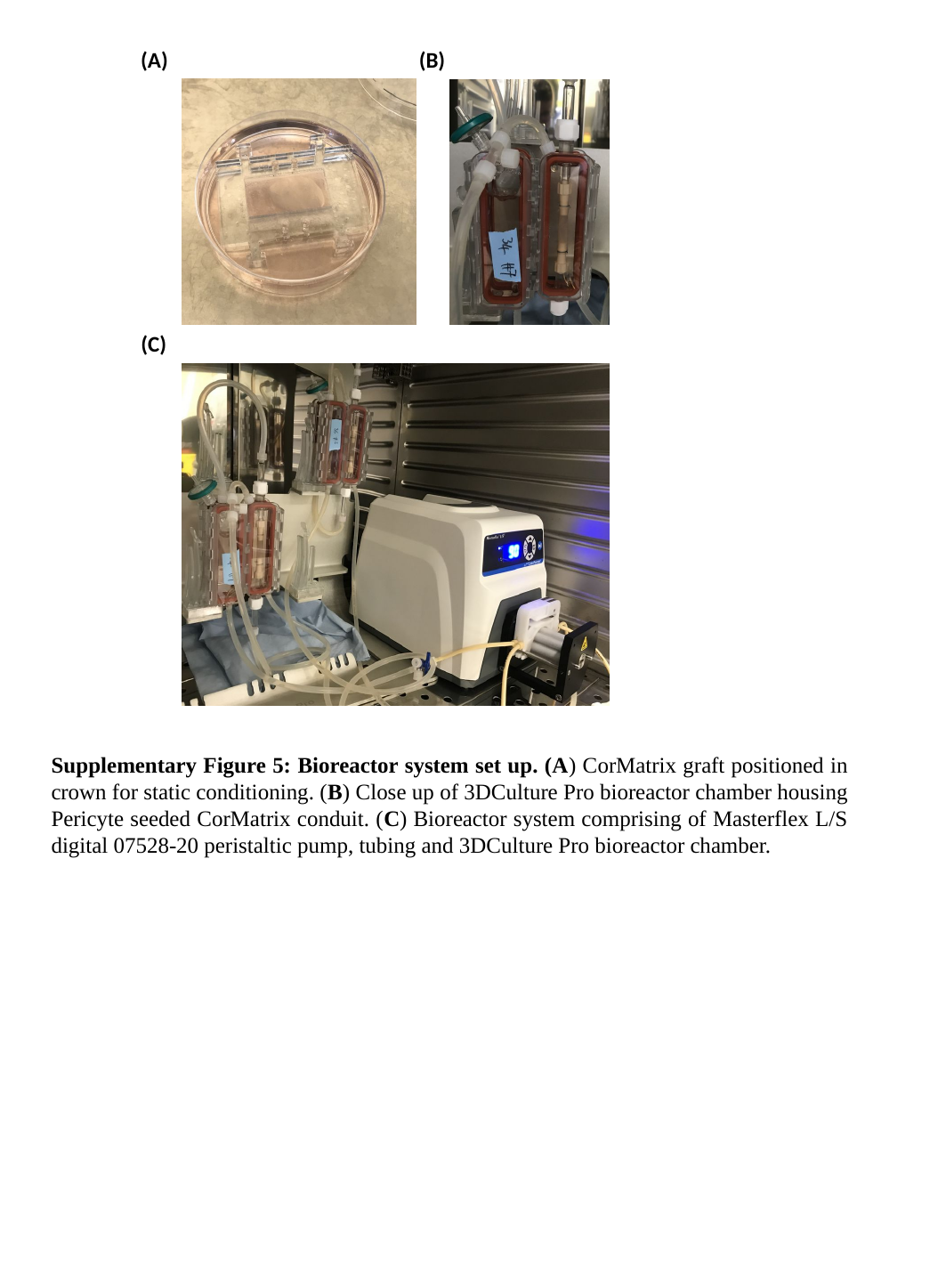

(A)
(B)
(C)
Supplementary Figure 5: Bioreactor system set up. (A) CorMatrix graft positioned in crown for static conditioning. (B) Close up of 3DCulture Pro bioreactor chamber housing Pericyte seeded CorMatrix conduit. (C) Bioreactor system comprising of Masterflex L/S digital 07528-20 peristaltic pump, tubing and 3DCulture Pro bioreactor chamber.
